# Network analysis reveals microbe-mediated impacts of aeration on deep sediment layer microbial communities

**DOI:** 10.3389/fmicb.2022.931585

**Published:** 2022-09-30

**Authors:** Zhenyu Wang, Feifei Liu, Enze Li, Yongqiang Yuan, Yonggang Yang, Meiying Xu, Rongliang Qiu

**Affiliations:** ^1^Guangdong Provincial Key Laboratory of Agricultural & Rural Pollution Abatement and Environmental Safety, College of Natural Resources and Environment, South China Agricultural University, Guangzhou, China; ^2^Guangdong Laboratory for Lingnan Modern Agriculture, Guangzhou, China; ^3^State Key Laboratory of Applied Microbiology Southern China, Guangdong Provincial Key Laboratory of Microbial Culture Collection and Application, Guangdong Institute of Microbiology, Guangdong Academy of Sciences, Guangzhou, China; ^4^Department of Biology, McMaster University, Hamilton, ON, Canada; ^5^Key Laboratory of Karst Georesources and Environment, Ministry of Education, College of Resources and Environmental Engineering, Guizhou University, Guiyang, China

**Keywords:** aeration, dissolved oxygen, phylogenetic microbial ecological networks, cable bacteria, river sediment

## Abstract

Over-aeration is a common remediation strategy for black and odorous water bodies, in which oxygen is introduced to impact aquatic microbial communities as an electron acceptor of high redox potential. In this study, black-odorous freshwater sediments were cultured for 9 weeks under aeration to investigate microbial covariations at different depths and time points. Based on community *16S rRNA* gene sequencing, the microbial covariations were visualized using phylogenetic microbial ecological networks (pMENs). In the spatial scale, we identified smaller and more compact pMENs across all layers compared with the anaerobic control sediments, in terms of network size, average node connectivity, and modularity. The aerated middle layer had the most connectors, the least module hubs, a network hub, shorter average path length, and predominantly positive covariations. In addition, a significant sulfate accumulation in the aerated middle layer indicated the most intense sulfide oxidation, possibly because aeration prompted sediment surface *Desulfobulbaceae*, known as cable bacteria, to reach the middle layer. In the time scale, similarly, aeration led to smaller pMEN sizes and higher portions of positive covariations. Therefore, we conclude that elevated dissolved oxygen at the water-sediment interface may impact not only the surface sediment but also the subsurface and/or deep sediment microbial communities mediated by microorganisms, particularly by *Desulfobulbaceae*.

## Introduction

Microbial ecosystems are responsible for the mitigation of greenhouse gas formation, carbon storage, removal of environmental pollutants, and supply of high-quality drinking water (Gruber and Galloway, 2008; Heathwaite, 2010). These functions are linked to biogeochemical element cycles driven by redox chemistry, with the electron donors and acceptors reacting either directly or through electron transport chains inside living cells (Borch et al., 2010; Karimian et al., 2018; Peiffer et al., 2021). In the water-sediment system, the poor penetration of dissolved oxygen (DO) into sediments results in a gradient content over the depth and, therefore, a depth-wise redox potential difference: an oxic sediment surface, a semi-oxic subsurface, and an anoxic deep sediment. This difference is responsible for shaping the community compositions and metabolisms of planktonic microorganisms and biofilms (Beman and Carolan, 2013; Mathe et al., 2014). Recent studies have revealed the ubiquitous presence of redox-active compounds in the water-sediment system, which are metastable (i.e., non-equilibrium solid phases), highly reactive, recyclable, and may act as biogeobatteries by storing electrons (Peiffer et al., 2021). For example, a low-level electrical current can be detected by connecting the deep sediment (where electron donors get oxidized) and the overlaying water (where DO gets reduced) using metal wires (Beman and Carolan, 2013).

Microbial extracellular electron transfer naturally occurs across the earth's hydrosphere stimulated by redox-active compounds, where a vast array of microorganisms produces electrical current via nanowire, nanotube, or fiber (Shi et al., 2016; Marzocchi et al., 2020). These electrochemically active microorganisms may serve as conduits transferring electrons from electron donors in the deep/anoxic sediment to electron acceptors in the upper oxic/suboxic layer (Ntarlagiannis et al., 2007). Particularly, cable bacteria that are members of the family *Desulfobulbaceae* and grow into electrically conductive multicellular filaments have been widely observed at the oxic–anoxic interface of marine and freshwater sediments (Risgaard-Petersen et al., 2015; Meysman, 2018). Thousands of cells line up in tandem, reaching down to ~3 cm in depth to bridge oxic and anoxic sediments. This “long-range” electron transport is believed more efficient than the nanowire networks (Nielsen and Risgaard-Petersen, 2015; Mueller et al., 2020). Mounting evidence indicates that cable bacteria may function as an electron sink for Fe(III) reducing or sulfur-oxidizing bacteria, exchanging electrons either by directly tapping onto each other or indirectly through electron shuttles (Vasquez-Cardenas et al., 2015; Otte et al., 2018). Furthermore, cable bacteria have similar effects as electrode snorkel in accelerating electron transfer and associated organic matter anaerobic degradation by harvesting electrons from the anoxic sediments and depositing them to oxygen or nitrate in the oxic sediments/overlaying water (Mueller et al., 2016; Marzocchi et al., 2020). Together, a network of microbe-microbe and microbe–mineral connections in sediment may function as cables, “hardwiring” the sediment and extending the impacts of surface water DO deep down. Due to the intrinsic complexity of benthic microbial communities, the network mechanism is still unclear.

Black-odorous water bodies are frequently reported in China, and over-aeration is a common remediation strategy for the rapid removal of ammonia, sulfide, and organic carbon (Cao et al., 2020; Huang et al., 2022). Sediments are massive reservoirs of various organic matter and nutrients (Lam et al., 2007; Kallmeyer et al., 2012; van Oevelen et al., 2013), hosting a large fraction of the earth's microbiome (Whitman et al., 1998; Wobus et al., 2003; Kallmeyer et al., 2012). Various sedimentary microbes interact through the exchange of electrons, nutrients, and information, forming complex ecological networks and affecting the associated ecological processes as a whole (Montoya et al., 2006). It is critical but challenging to correlate microbial interactions and over-aeration (Raes et al., 2007; Zhou et al., 2010). Microbial network analysis is suitable for retrieving community structure and interactions from large environmental datasets (Steele et al., 2011; Zhou et al., 2011; Cordero and Datta, 2016; Feng et al., 2017; Layeghifard et al., 2017). Microbial interactions and co-occurrence patterns are characterized by two network components: nodes and links. Depending on the types of omics data, nodes can be any biological units of interest such as microbial taxa, genes, and proteins, whereas links represent the statistically significant covariations among nodes. Furthermore, topological analysis of networks allows the prediction of hub biological units that presumably contribute most to outlining community structure and functions (Banerjee et al., 2016; Layeghifard et al., 2017; Rottjers and Faust, 2018). Additionally, by finding similarities and discrepancies in the topologies of microbial networks subject to different environments, network analysis has the potential to provide insights into the alteration of species/genes interactions under over-aeration conditions (Zhou et al., 2011; Feng et al., 2017). Thus, the network approach provides an unprecedented opportunity to characterize the influence of surface water DO fluctuation caused by over-aeration on benthic microbial communities which is likely to reach deeper sediments *via* cable bacteria and other microorganisms (Liu et al., 2021).

In this study, black-odorous river sediments were collected and aerated under laboratory conditions over 9 weeks. The community composition was measured using high-throughput sequencing of *16S rRNA* genes, upon which phylogenetic microbial ecological networks (pMENs) were constructed. We aim to profile the depth-wise and temporal correlations between sediment microbial covariations and dissolved oxygen disturbance, to address (i) how sediment surface DO would influence sediment microbial communities with respect to compositions, functions, and covariations; (ii) how deep this influence could reach into the sediments; and (iii) how the influence would change over time. We hypothesize that elevated DO would promote the growth of electrochemically active sediment microorganisms such as cable bacteria, which in turn would facilitate introducing DO influences to deeper sediments, such as extending the microbial respiratory chain as a terminal electron acceptor and, therefore, alter microbial covariations. Consequently, these microbial mediators would form predominantly positive covariations with other existing taxa whose electron transport chain can be extended by DO.

## Materials and methods

### Experimental setup

The subject sediment was collected in September 2020 from a creek in the Pearl River Delta (22°45′31.7″N, 113°16′22.0″E), where the presence of cable bacteria was visually confirmed in our previous study (Liu et al., 2021). Briefly, a metal sediment dredging sampler fastened with a rope was settled down to the sediment surface; then, ~5 L sediment (0–30 cm in depth) was grabbed inside the sampler. During the sediment collection, the natural sediment stratification was disrupted. Sampled sediment was kept on ice and transported to the laboratory. In the laboratory, sampled sediments were sieved through a 50-mesh filter (pore size = 0.35 mm, diameter = 30 cm, Changzhou Feilong filter Inc., China) to remove rocks and other miscellaneous debris and were homogenized.

Lab incubation was carried out in 60 beakers (100 ml) at room temperature, with each beaker filled with 80 ml (~120 g) of sediment as microbial inoculum. All beakers were submerged in two rectangle reservoirs filled with river water, one as a treatment group and the other as a control. Aeration was continuously applied at the four corners of the treatment reservoir to avoid disturbing the sediments. Deionized water was replenished every 48 h following water evaporation. The reason we replenished deionized water was to avoid introducing new ions to the incubation reservoirs and to be in accordance with our previous study (Hu et al., 2021; Liu et al., 2021); this, however, would inevitably dilute the existing ions and water-soluble chemicals in the river water.

According to Schauer et al. (2014) and Burdorf et al. (2018), the abundance of *Desulfobulbaceae* peaks within 2–4 weeks after sediment disturbance and then declines possibly due to the depletion of electron donors, such as reduced sulfur compounds. Therefore, the total incubation lasted 9 weeks, with micro-profiles recorded at the end of Weeks 1, 2, 3, 4, and 9 to closely monitor the thriving of *Desulfobulbaceae* and the tendency thereafter. At each time point, six beakers from the control and treatment reservoir were removed and subsampled vertically in three layers (0–2 cm as the top layer, 2–4 cm as the middle layer, and 4–6 cm as the bottom layer) for DNA extractions and chemical measurements.

### Chemical analyses

Three groups of physiochemical parameters were measured: (i) the concentrations of ammonium and sulfate in the overlying water were analyzed using ion chromatography (DIONEX ICS-1100) equipped with a Dionex Ion Pac AS25 column (4 × 250 mm); (ii) total carbon (TC), total organic carbon (TOC), and total nitrogen (TN) in liquid and solid phases were analyzed with a TOC analyzer (TOC-L, SHIMADZU Corp., Japan); (iii) available phosphorus in sediments was measured according to Li et al. (2018).

### DNA isolation and sequencing

Microbial genomic DNA was isolated using DNeasy PowerSoil Isolation Kit (QIAGEN, Germany). DNA quality and quantity were recorded using a UV spectrophotometer (NanoDrop One^C^, Thermo Fisher). V3–V4 hypervariable region of the *16S rRNA* gene was amplified using barcoded 338F (5′-ACTCCTACGGGAGGCAGCA-3′) and 806R (5′-GGACTACHVGGGTWTCTAAT-3′) primer pair (Lee et al., 2012). For each sediment sample, PCR was conducted in triplicate, each 25 μl reaction contained 0.5 U Taq DNA polymerase (TaKaRa Inc., Japan), 2.5 μl 10 × PCR buffer, dNTPs (75 nM each), 10 μM forward and reverse primers each, 100 ng template DNA, and ddH_2_O. PCR protocol included an initial denaturation (98°C, 3 min), 30 cycles at 98°C for 30 s, 50°C for 30 s, and 72°C for 1 min, and a final extension (72°C, 5 min). PCR products were examined using agarose gel electrophoresis. Triplicate PCR products were pooled together and subject to Qubit quantification (Thermo Fisher, USA). Thereafter, 200 ng of PCR products from each sediment was pooled and purified with a QIAquick Gel Extraction Kit (QIAGEN). The purified mixture was diluted to 2 nM for library construction according to MiSeq Reagent Kit Preparation Guide (Illumina). Constructed libraries were sequenced on HiSeq 2500 (Illumina).

### Sequence data analyses

Raw amplicon sequencing reads are available in the National Omics Data Encyclopedia (NODE) with the project number OEP001302 (https://www.biosino.org/node/). Raw reads were processed in the QIIME2 platform (Caporaso et al., 2010). First, they were demultiplexed using the q2-demux plugin (https://github.com/qiime2/q2-demux); then, forward and reverse reads were trimmed at 220 and 180 base pairs, respectively. Paired reads were merged, quality filtered, dereplicated, and removed from chimeric sequences with the q2-dada2 plugin (https://benjjneb.github.io/dada2/) (Callahan et al., 2016, 2017). Amplicon sequence variants (ASVs) were generated using default parameters in DADA2. The resulting ASVs were assigned a taxonomic classification using the Silva *16S rRNA* gene database (https://www.arb-silva.de/) and QIIME2 q2-feature-classifier (https://github.com/qiime2/q2-feature-classifier) (Bokulich et al., 2016). By default, ASVs less than 8 counts across all 180 samples were automatically filtered in the denoising step. For bacterial community analyses, we rarefied each sample to a sequencing depth of 48,000 reads and removed ASVs below 0.001% relative abundance to reduce the computational burden. Note that it is generally not recommended to further filter data after rarefaction, though many ASVs may turn into lower total counts or even complete absence. This sequencing depth was considered acceptable according to the rarefaction curves in terms of observed ASV numbers (i.e., richness) and Shannon index (Supplementary Figure S1).

### Network construction and characterization

To explore the effects of over-aeration on the sediment microbial interactions, pMENs for both aerated and control sediments at different depths across the incubation period were constructed based on the random matrix theory and the molecular ecological network analysis pipeline (MENA, http://ieg4.rccc.ou.edu/mena/main.cgi) (Shannon et al., 2003; Zhou et al., 2010; Deng et al., 2012, 2016; Liang et al., 2016; Feng et al., 2017). Briefly, only ASVs present in more than half of the total samples of each group were kept; the remaining data matrix was subject to algorithm transformation and calculation of Spearman's Rho rank correlation coefficient:


$$r_{s}\;=\;1-\frac{6\sum d_{i}^{2}}{n(n^{2}-1)}\;(\text{depreciated}),$$

where rg_*x*_, rg_*y*_ is the rank of the raw score, d_*i*_ = rg(X_*i*_)–rg(Y_*i*_) is the difference, and *n* is the number of observations. *r*_*s*_ thresholds from 0.01 to 0.95 with a 0.01 increment were applied to the matrix to calculate network eigenvalues. The most suitable threshold was determined when the nearest-neighbor spacing showed a good Poisson distribution, where constructed pMENs were to reflect specific and non-random properties of a complex system (Luo et al., 2007). Unless stated otherwise, the Spearman correlation coefficient threshold of 0.83 was selected to generate pMENs to compare topological characteristics of different networks under the same conditions, with respect to the number of nodes and links, average connectivity, average path length, average clustering coefficient, proportion of positive links, and modularity (Deng et al., 2012). Constructed networks were visualized using Cytoscape (v3.9.1) (Bastian et al., 2009).

A module refers to a group of nodes that have more connections with each other than with nodes outside the group. In MENA, there are four built-in methods for module separation, and we selected the default greedy modularity optimization because it generates the highest modularity index (M), i.e., more effective in separating a network into submodules (Deng et al., 2012). Node connectivity in a pMEN reflects ASV ecological roles in a microbial community (Guimera et al., 2007). Here, the connectivity of individual nodes in the depth-wise and temporal pMENs was determined based on the within-module connectivity (*Z*_*i*_) and among-module connectivity (*P*_*i*_). In the *ZP* biplot, nodes were categorized into four roles: (i) peripherals (*Z*_*i*_ ≤ 2.5, *P*_*i*_ ≤ 0.62) are nodes with no significant connectivity, and their connections are mostly within their own modules; (ii) module hubs (*Z*_*i*_ > 2.5, *P*_*i*_ ≤ 0.62), heavily connected to nodes within their own modules; (iii) connectors (*Z*_*i*_ ≤ 2.5, *P*_*i*_ > 0.62), heavily connected to nodes outside their own modules; and (iv) network hubs that are both module hubs and connectors (Olesen et al., 2007). Module hubs, connectors, and network hubs are classified as keystone nodes.

## Results

### Sediment chemical property changes in response to aeration

Dissolved oxygen content at the sediment-water interface of control sediments was 2.90 mg/L, which decreased over depth and became undetectable at ~2 mm beneath the interface. Under aeration, DO content at the interface was maintained at 7.80 mg/L, ~2.7 times that in the control sediments (Table 1). Similarly, it dropped quickly over depth and became undetectable at ~5 mm. This meant only the top layer (0–2 cm) was directly exposed to DO. Sulfate accumulations were observed across all layers of the aerated sediments during the first 4 weeks, and the most intensive sulfate accumulation occurred in the middle layer (2–4 cm), from 56.56 ± 1.87 mg/L (Week 1) to 219.18 ± 6.51 mg/L (Week4) (Supplementary Figure S2). Consistently higher [ammonium] was observed in control than in the aerated sediments at all times regardless of sediment depth, except for the Week 1-middle layer and Week 3-bottom layer. Furthermore, [ammonia] of different layers of both aerated and control sediments were significantly related to depths (Spearman's Rho rank correlation coefficient *r*_*s*_ = 0.838, *P* = 0.001 for aerated sediment and *r*_*s*_ = 0.812, *P* = 0.0007 for control sediment). Total carbon, total organic carbon, and total nitrogen contents in the sediment were not significantly affected by aeration regardless of sediment depths (*P* = 0.051–0.754 by paired *t*-test). In contrast, aeration significantly reduced total nitrogen content in the porewater above the top layer (*P* = 0.012).

**Table 1:** Chemical properties of porewater and sediments.

		**Aeration**					**Control**				
		**Week 1**	**Week 2**	**Week 3**	**Week 4**	**Week 9**	**Week 1**	**Week 2**	**Week 3**	**Week 4**	**Week 9**
Top layer	pH	7.20 ± 0.02	6.71 ± 0.03	6.65 ± 0.03	6.66 ± 0.01	7.00 ± 0.04	7.53 ± 0.08	7.35 ± 0.05	7.30 ± 0.08	7.19 ± 0.04	7.07 ± 0.03
	[SO${}_{4}^{2-}$]	27.68 ± 4.70	131.48 ± 19.97	143.12 ± 13.51	181.34 ± 9.76	37.3 ± 2.48	15.09 ± 2.51	22.24 ± 3.30	21.32 ± 3.45	22.48 ± 2.84	11.82 ± 1.99
	[NH${}_{4}^{+}$]	9.19 ± 0.97	6.15 ± 0.72	6.05 ± 0.29	6.36 ± 0.31	3.09 ± 0.37	17.44 ± 2.03	16.49 ± 2.06	15.63 ± 1.09	14.88 ± 1.68	10.01 ± 0.59
	pTN	41.20 ± 3.49	30.27 ± 2.51	18.49 ± 4.12	11.19 ± 0.75	9.87 ± 0.52	79.18 ± 2.00	53.23 ± 3.33	32.03 ± 3.32	29.94 ± 0.63	20.61 ± 2.96
	pTC	54.79 ± 1.53	76.81 ± 0.69	41.23 ± 2.48	40.56 ± 0.86	38.95 ± 1.72	77.09 ± 2.66	77.40 ± 2.33	48.10 ± 4.51	37.42 ± 1.78	38.22 ± 4.49
	pTOC	21.90 ± 2.08	20.51 ± 0.30	18.03 ± 1.86	15.14 ± 0.25	8.28 ± 0.33	31.95 ± 1.06	19.26 ± 1.71	17.48 ± 1.42	18.83 ± 3.19	14.46 ± 1.35
	sTN	4.25 ± 0.15	4.26 ± 0.13	3.99 ± 0.25	4.10 ± 0.13	4.19 ± 0.41	4.29 ± 0.12	4.23 ± 0.10	4.03 ± 0.15	4.19 ± 0.16	4.24 ± 0.34
	sTC	46.61 ± 4.39	48.92 ± 3.10	44.19 ± 2.85	40.81 ± 5.60	36.96 ± 8.34	46.12 ± 2.36	45.58 ± 3.44	43.79 ± 6.63	42.41 ± 4.48	37.48 ± 3.82
	sTOC	45.10 ± 4.08	47.92 ± 3.15	43.26 ± 3.39	40.13 ± 5.35	34.35 ± 6.16	44.05 ± 3.02	44.26 ± 3.50	42.40 ± 6.76	41.65 ± 4.64	35.94 ± 3.84
	sAP	96.48 ± 2.93	88.92 ± 1.95	81.16 ± 2.55	79.64 ± 1.49	78.64 ± 3.55	78.80 ± 1.70	77.84 ± 5.33	86.28 ± 1.16	77.84 ± 1.83	86.32 ± 1.63
Middle layer	pH	7.40 ± 0.07	7.05 ± 0.05	6.59 ± 0.04	6.01 ± 0.01	6.27 ± 0.03	7.37 ± 0.06	7.28 ± 0.05	7.37 ± 0.08	7.32 ± 0.04	7.18 ± 0.03
	SO${}_{4}^{2-}$	58.56 ± 1.87	157.75 ± 14.22	192.38 ± 8.75	219.18 ± 6.51	67.97 ± 9.41	1.44 ± 0.32	1.36 ± 0.63	3.02 ± 0.62	2.88 ± 0.95	3.34 ± 0.61
	NH${}_{4}^{+}$	8.79 ± 0.22	14.43 ± 1.31	18.65 ± 1.32	16.78 ± 0.50	3.78 ± 0.54	5.37 ± 0.75	17.03 ± 1.54	23.03 ± 1.98	20.95 ± 2.60	13.46 ± 0.85
	pTN	103.29 ± 7.54	81.72 ± 1.47	44.23 ± 8.21	33.25 ± 5.33	12.72 ± 1.82	100.71 ± 3.13	79.42 ± 1.73	44.28 ± 6.24	44.27 ± 3.43	28.44 ± 3.74
	pTC	72.82 ± 2.82	64.05 ± 4.27	52.25 ± 4.73	34.74 ± 4.69	35.34 ± 2.26	83.65 ± 3.47	74.66 ± 0.65	59.04 ± 5.14	56.92 ± 2.45	49.77 ± 8.05
	pTOC	20.64 ± 1.85	18.21 ± 0.48	14.84 ± 0.44	12.28 ± 1.70	9.56 ± 0.69	20.35 ± 1.09	19.19 ± 0.34	20.50 ± 1.00	17.36 ± 3.22	18.78 ± 4.20
	sTN	4.40 ± 0.14	4.19 ± 0.15	4.15 ± 0.07	4.25 ± 0.16	4.29 ± 0.36	4.29 ± 0.13	4.37 ± 0.27	4.07 ± 0.40	4.30 ± 0.09	4.15 ± 0.12
	sTC	50.52 ± 3.47	46.06 ± 3.17	42.17 ± 7.17	45.06 ± 3.17	42.41 ± 4.48	50.64 ± 6.31	47.42 ± 5.00	47.44 ± 4.08	47.55 ± 8.78	39.12 ± 4.32
	sTOC	48.82 ± 3.58	44.81 ± 3.63	40.85 ± 7.14	42.89 ± 2.83	41.65 ± 4.64	48.98 ± 6.37	45.90 ± 4.93	45.80 ± 3.93	44.48 ± 8.07	37.33 ± 4.72
	sAP	85.92 ± 1.27	83.20 ± 2.96	79.00 ± 4.43	75.44 ± 1.23	73.52 ± 1.09	79.28 ± 1.63	78.56 ± 1.85	83.88 ± 1.43	77.64 ± 1.07	73.80 ± 2.26
Bottom layer	pH	7.48 ± 0.05	7.25 ± 0.04	6.95 ± 0.02	6.54 ± 0.04	6.61 ± 0.02	7.30 ± 0.06	7.28 ± 0.03	7.26 ± 0.04	7.27 ± 0.04	7.21 ± 0.04
	SO${}_{4}^{2-}$	27.26 ± 4.85	81.87 ± 15.41	109.43 ± 17.09	132.04 ± 19.70	30.29 ± 4.01	1.18 ± 0.48	2.97 ± 0.91	3.24 ± 1.09	2.16 ± 0.60	1.61 ± 0.33
	NH${}_{4}^{+}$	4.14 ± 0.55	15.68 ± 1.44	30.21 ± 1.17	24.20 ± 1.40	7.88 ± 0.69	26.44 ± 2.35	26.39 ± 1.67	28.92 ± 1.13	28.14 ± 0.80	11.29 ± 0.98
	pTN	136.01 ± 12.68	102.80 ± 3.98	71.32 ± 7.39	40.99 ± 7.55	19.99 ± 0.82	131.99 ± 4.98	103.88 ± 2.60	64.43 ± 3.34	66.73 ± 2.44	38.29 ± 1.89
	pTC	90.62 ± 3.45	103.36 ± 5.38	81.65 ± 5.36	62.24 ± 4.12	44.47 ± 3.18	111.94 ± 6.47	60.70 ± 1.72	76.04 ± 3.57	75.72 ± 2.10	51.90 ± 1.71
	pTOC	28.04 ± 2.13	25.89 ± 1.64	20.89 ± 0.53	17.82 ± 1.68	12.01 ± 0.19	26.91 ± 0.42	21.41 ± 1.99	20.37 ± 0.72	18.81 ± 1.34	10.79 ± 0.19
	sTN	4.40 ± 0.18	4.26 ± 0.06	4.14 ± 0.11	4.12 ± 0.10	3.83 ± 0.30	4.38 ± 0.18	4.35 ± 0.1	4.26 ± 0.08	4.29 ± 0.26	4.07 ± 0.08
	sTC	50.95 ± 4.58	44.34 ± 1.28	49.18 ± 3.37	43.68 ± 6.11	36.29 ± 6.22	54.22 ± 6.89	46.15 ± 1.43	50.43 ± 5.74	41.89 ± 4.21	42.19 ± 1.01
	sTOC	49.50 ± 4.68	42.78 ± 1.35	47.30 ± 3.86	41.58 ± 6.26	34.42 ± 6.14	52.32 ± 7.03	44.30 ± 1.63	48.40 ± 5.19	39.85 ± 3.94	40.60 ± 0.85
	sAP	82.32 ± 2.65	85.16 ± 4.74	85.84 ± 3.79	75.24 ± 1.29	75.00 ± 4.45	81.08 ± 3.24	81.16 ± 2.82	87.44 ± 1.7	80.04 ± 2.76	77.32 ± 1.64

Each value was averaged from 5 parallel measurements and showed as mean ± (standard deviation).

sTN, sediment total nitrogen, mg/g sediment; sTC, sediment total carbon, mg/g sediment; sTOC, sediment total organic carbon, mg/g sediment; sAP, sediment total available phosphorus, μg/g sediment; pTN, porewater total nitrogen; pTC, porewater total carbon; pTOC, porewater total organic carbon; pAP, porewater total available phosphorus; [SO${}_{4}^{2-}$] and [NH${}_{4}^{+}$] in mg/L.

### Aeration shaped unique top layer microbial communities

Prior to network analysis, we compared the compositions of microbial communities at the phylum and genus level, between control and aerated sediments (Supplementary Figure S3). Among the 11 predominant phyla (>1.0% relative abundance), *Proteobacteria* in the top layer showed significant enrichment in response to aeration throughout the 9-week incubation, whereas *Proteobacteria* in the middle and bottom layers did not. Approximately two-thirds of the total ASVs were not assigned to a specific genus. Among the 17 predominant genera (>0.5% relative abundance), *Clostridium sensu stricto* 1, *Romboutsia, Caldisericum*, and *Smithella* ranked the most abundant. The non-metric multidimensional scaling based on Bray–Curtis dissimilarity showed that both incubation and aeration impacted the microbial compositions (Supplementary Figure S4). However, the aerated top layers had the most distinct microbial compositions among all other layers throughout the incubation. This reconciled our observation since only the top layer was directly exposed to DO, and the aerated top layer had ~2.7 times DO content than the control top layer.

### Depth-wise effects of aeration on sediment microbial pMENs

**Figure 1:**
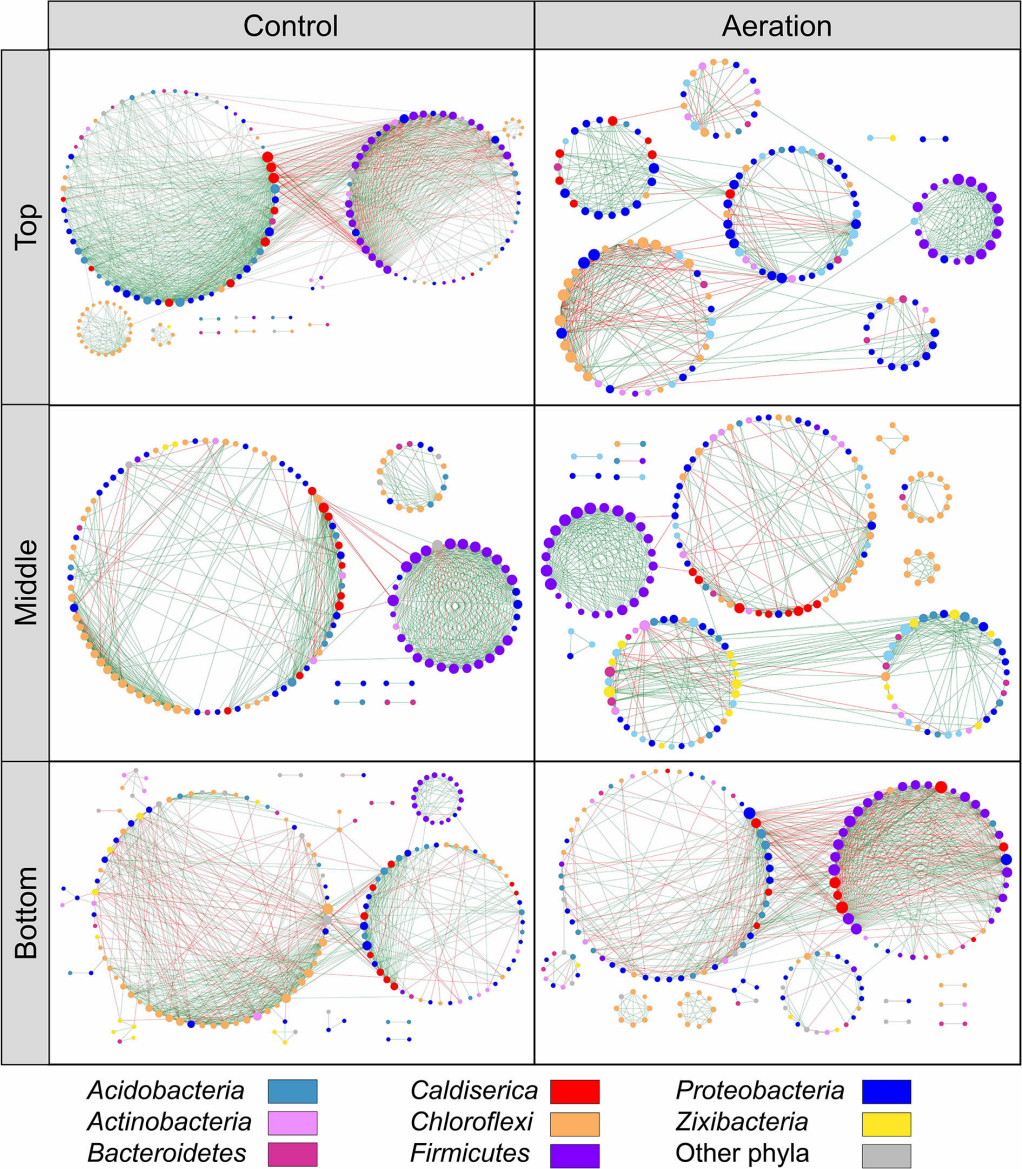
Depth-wise pMENs showing significant abundance covariations of sediment microorganisms at the top, middle, and bottom layers under aeration. Each node represented an ASV in different modules. Major contributing phyla were colored. Node size was proportional to node degree. A link represented a significant Spearman's Rho rank correlation between two nodes, green for positive and red for negative correlations.

We generated six depth-wise empirical pMENs based on the ASV covariations of the top, middle, and bottom layer sediment microbial communities from the aeration or control group (Figure 1). In these pMENs, each node represented an ASV displayed at the phylum level, showing significant pairwise covariations with at least one node. All pMENs exhibited typical scale-free, small-world, and modularity characteristics as indicated by the well-fitted power law model (0.828 < R^2^ < 0.934) (Table 2) (Deng et al., 2012). Differences with respect to composition and connectivity between aerated and control pMENs were found. For example, aerated pMENs had consistently higher modularity than control pMENs across all layers, and only 132 (24.1%), 163 (31.5%), and 151 (31.7%) nodes constituting each network pair were shared by the two groups at the top, middle, and bottom layer. These shared nodes belonged to 22 phyla among which *Chloroflexi, Proteobacteria, Bacteroidetes*, and *Firmicutes* were the most abundant (Supplementary Table S1). When comparing the top layers, the major phyla contributing to microbial covariations (with respect to average node connectivity) were *Firmicutes* (10.3, *n* = 19), *Caldiserica* (8.7, *n* = 7), and *Chloroflexi* (8.0, *n* = 33) for the aerated group and were *Caldiserica* (34.0, *n* = 10), *Firmicutes* (23.2, *n* = 29), and *Proteobacteria* (16.4, *n* = 32) for the control group. Therefore, phylum abundances in pMENs were not necessarily indicative of phylum connectivity. Moreover, positive link proportions were 12.31–22.26% higher in aerated sediments across all layers than in control sediments.

**Table 2:** Topological properties of the empirical and associated random pMENs of microbial communities at different sediment layers under aeration and control.

		**Empirical networks**								**Random networks** ^**a**^		
**Communities**		**Similarity threshold**	**Network size**	**R^2^ of power law**	**Avg. connectivity**	**Positive links**	**Avg. path length^b^**	**Avg. clustering coefficient**	**Modularity**	**Avg. path length^b^**	**Avg. clustering coefficient**	**Modularity**
Top layer	Aeration	0.83	313	0.887	4.921	72.5%	4.437	0.256	0.692	3.464 ± 0.037	0.053 ± 0.008	0.406 ± 0.006
	Control	0.83	367	0.828	5.003	64.5%	5.449	0.208	0.587	3.446 ± 0.045	0.072 ± 0.009	0.398 ± 0.005
Middle layer	Aeration	0.83	324	0.858	4.154	60.2%	5.063	0.222	0.724	3.644 ± 0.047	0.048 ± 0.008	0.467 ± 0.007
	Control	0.83	357	0.912	4.252	53.6%	5.298	0.203	0.712	3.700 ± 0.046	0.039 ± 0.007	0.465 ± 0.006
Bottom layer	Aeration	0.83	271	0.878	3.358	78.0%	6.174	0.194	0.711	4.133 ± 0.066	0.023 ±0.006	0.555 ± 0.008
	Control	0.83	358	0.934	4.045	63.8%	5.650	0.212	0.667	3.816 ± 0.051	0.034 ± 0.006	0.485 ± 0.006

^a^Random networks were generated by rewiring all nodes and links corresponding to empirical networks, bootstrap = 100. ^b^Determined by geodesic distance.

pMENs-associated random networks were also generated to evaluate the statistical significance of network properties (Table 2). Consistently smaller network size and average connectivity which reflected the lower complexity were observed in aerated sediments, suggesting simplified networks. Compared to control, the shorter average path length and higher average clustering coefficient for the aerated top and middle layer pMENs suggested tighter node connections.

### Temporal effects of aeration on sediment microbial pMENs

**Figure 2:**
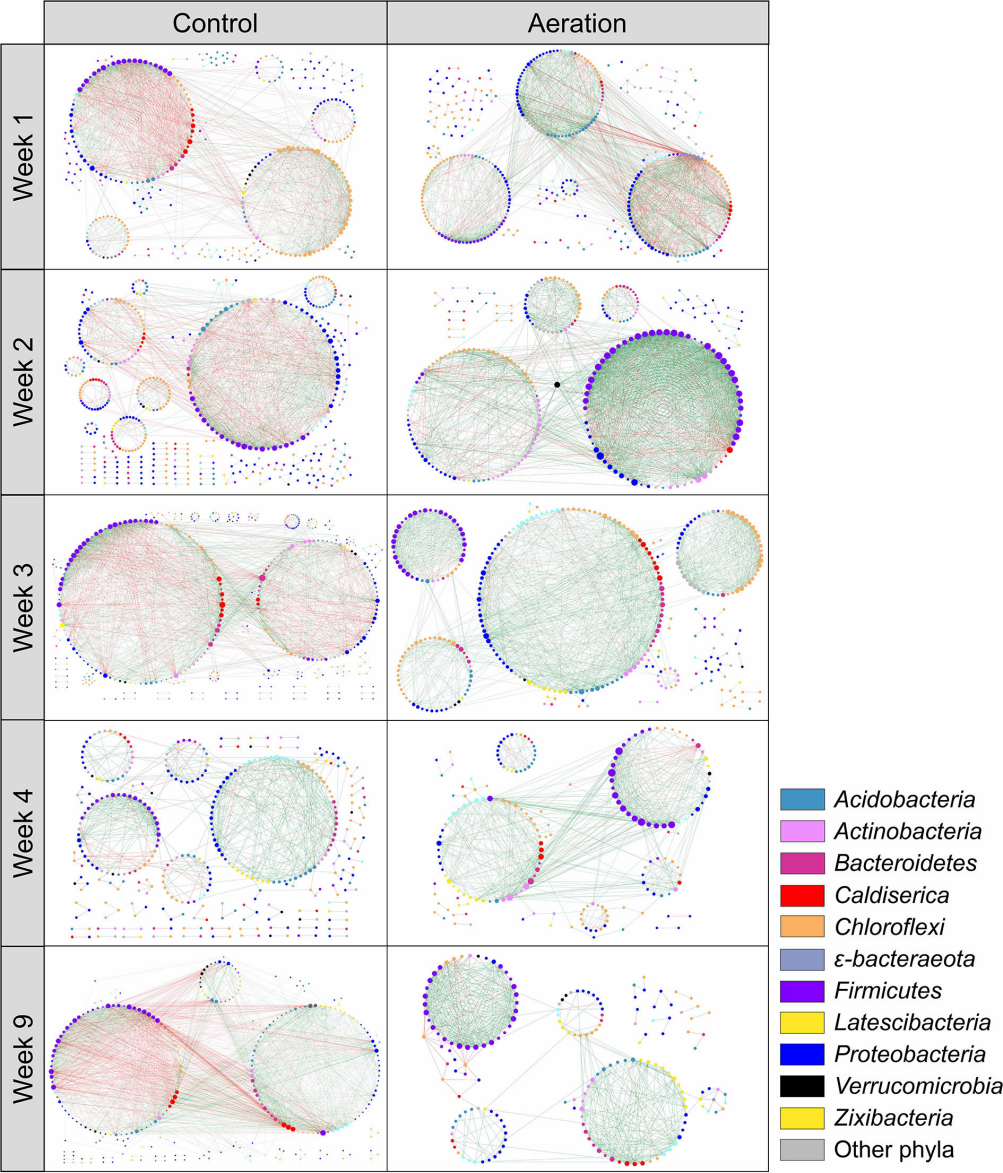
Temporal pMENs showing significant abundance covariations of sediment microorganisms at five sampling times during the 9-week incubation under aeration. Each node represented an ASV in different modules. Major contributing phyla were colored. Node size was proportional to node degree. A link represented a significant Spearman's Rho rank correlation between two nodes, green for positive and red for negative correlations.

Sediment microbial communities of all layers at the same time point were combined to generate temporal pMENs to examine network variations over time (Figure 2). Network metrics are shown in Table 3. Aerated temporal pMENs had consistently higher average connectivity, higher average clustering coefficient, shorter average path length, and higher proportions of positive links, indicative of tighter and more cooperative networks. In contrast, network sizes of aerated pMENs were consistently lower than control pMENs and kept decreasing over time. Additionally, the positive link proportion gradually increased over time and peaked at Week 3 (78.5%) and then remained steady thereafter. Similar to depth-wise pMENs, phylum abundances in temporal pMENs were not necessarily indicative of phylum connectivity as well. For example, at the end of the incubation (Week 9), the major microbial covariation contributing phyla in aerated pMEN were *Firmicutes* (13.4, *n* = 31), *Caldiserica* (8.9, *n* = 7), and *Bacteroidetes* (6.5, *n* = 11) and were *Caldiserica* (50, *n* = 8), *Firmicutes* (32.6, *n* = 40), and *Actinobacteria* (18.0, *n* = 8) in control pMEN.

**Table 3:** Topological properties of the empirical and associated random phylogenetic molecular ecological networks of sediment microbial communities under aeration and control over time.

		**Empirical networks**								**Random networks** ^**a**^		
**Communities**		**Similarity threshold**	**Network size**	**R^2^ of power law**	**Avg. connectivity**	**Positive edge proportion**	**Avg. path length^a^**	**Avg. clustering coefficient**	**Modularity**	**Avg. path length^a^**	**Avg. clustering coefficient**	**Modularity**
Aeration	Week 1	0.81	499	0.903	5.347	57.8%	5.500	0.166	0.573	3.502 ± 0.031	0.055 ± 0.006	0.383 ± 0.005
	Week 2	0.83	445	0.785	7.604	77.2%	4.709	0.222	0.498	3.134 ± 0.029	0.113 ± 0.008	0.274 ± 0.005
	Week 3	0.85	361	0.865	4.654	78.5%	6.323	0.230	0.712	3.588 ± 0.039	0.036 ± 0.007	0.429 ± 0.005
	Week 4	0.85	375	0.892	5.952	78.0%	5.099	0.168	0.441	3.276 ± 0.035	0.085 ± 0.007	0.343 ± 0.005
	Week 9	0.87	323	0.818	6.236	73.7%	5.972	0.180	0.674	3.565 ± 0.046	0.049 ± 0.008	0.438 ± 0.007
Control	Week 1	0.82	517	0.834	4.081	50.3%	8.897	0.133	0.685	3.757 ± 0.046	0.043 ± 0.006	0.466 ± 0.006
	Week 2	0.81	497	0.926	2.592	60.7%	7.162	0.077	0.787	4.782 ± 0.092	0.012 ± 0.004	0.682 ± 0.006
	Week 3	0.82	484	0.912	3.140	51.2%	6.383	0.091	0.715	4.320 ± 0.060	0.019 ± 0.005	0.591 ± 0.006
	Week 4	0.81	514	0.924	2.113	59.8%	9.515	0.050	0.857	6.022 ± 0.208	0.005 ± 0.003	0.797 ± 0.008
	Week 9	0.83	492	0.788	4.421	55.4%	6.161	0.156	0.418	3.248 ± 0.030	0.120 ± 0.009	0.314 ± 0.005

^a^Random networks were generated by rewiring all nodes and links corresponding to empirical networks, bootstrap = 100. ^b^Determined by geodesic distance.

### Variation in the connectivity of individual nodes in response to aeration

**Figure 3:**
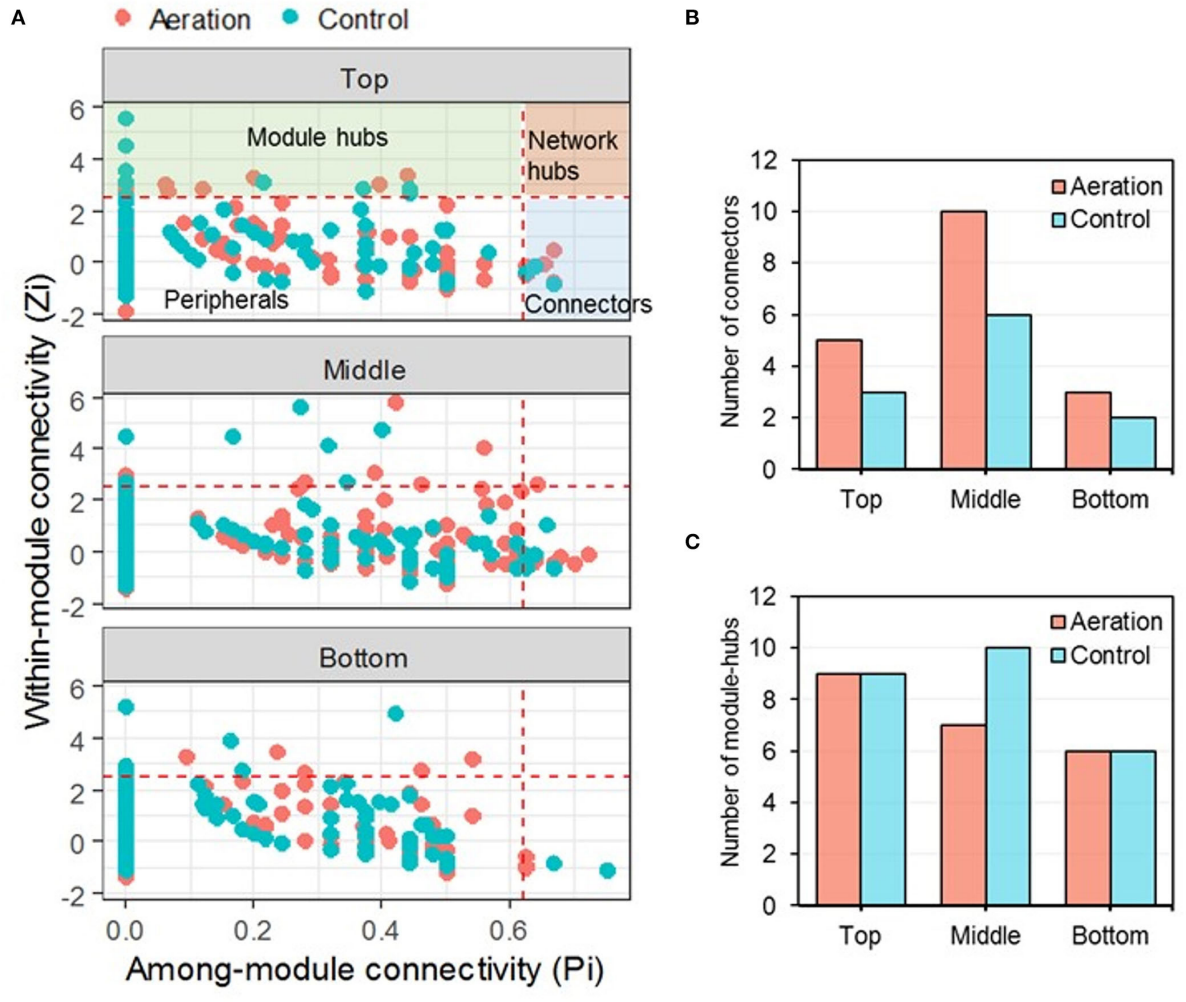
*ZP* plot of depth-wise pMENs showing the distribution of nodes based on their within-module (*Z*_*i*_) and among-module (*P*_*i*_) connectivity **(A)** together with the numbers of keystone nodes, including connectors **(B)** and module hubs **(C)**. One node (ASV_61, member of the family *Anaerolineaceae*) was classified as a network hub present in the aerated middle pMEN.

For depth-wise pMENs, the majority (94.8–97.8%) of the nodes were peripherals, indicating that predominant connections were established within modules (Figure 3A). There were 29 connectors in total, 18 in aerated pMENs, and 11 in control pMENs. Aerated pMENs had more connectors than control pMENs across all layers, with the biggest difference found in the middle layer (Figure 3B). Interestingly, ASV_712 (family *Anaerolineaceae*, phylum *Chloroflexi*) was the connector of both aerated bottom and control bottom pMENs (Supplementary Table S2). Among the total 47 module hubs, 22 were in aerated pMENs and 25 in control (Supplementary Table S3). Aeration selectively reduced the number of module hubs of the middle layer, leaving the top and bottom layers less affected (Figure 3C). Furthermore, three module hubs (ASV_20, ASV_63, and ASV_663, members of phylum *Acidobacteria, Chloroflexi*, and *Verrucomicrobia*, respectively) were shared by aerated and control pMENs. Within the aerated pMENs, ASV_6 (genus *Caldisericum*) appeared in all three layers as a module hub, whereas another three module hubs (ASV_28, ASV_144, and ASV_36, members of the family *Lentimicrobiaceae, Xanthobacteraceae*, and *Pedosphaeraceae*, respectively) appeared in at least two layers of control pMENs. In addition, one network hub (ASV_61, family *Anaerolineaceae*, phylum *Chloroflexi*) was identified in the aerated middle pMEN.

**Figure 4:**
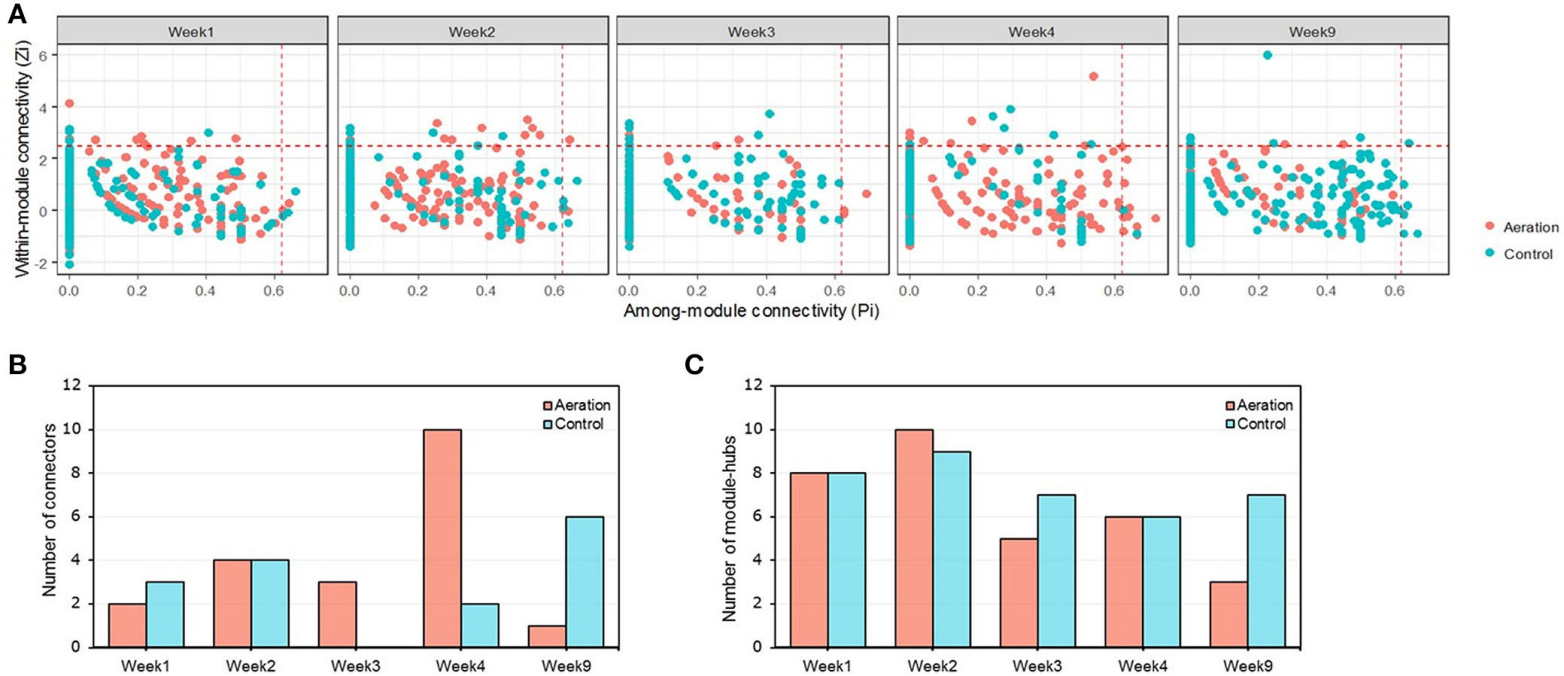
*ZP* plot of temporal pMENs showing the distribution of nodes based on their within-module (*Z*_*i*_) and among-module (*P*_*i*_) connectivity **(A)** together with the numbers of keystone nodes, including connectors **(B)** and module hubs **(C)**. Two nodes (ASV_144, member of family *Sphingomonadaceae*, and ASV_572, member of family *Pedosphaeraceae*) were classified as network hubs present in the control Week 9 and aerated Week 2 pMENs.

Changes in node connectivity among the temporal pMENs in response to aeration resembled those among depth-wise pMENs. Specifically, aerated pMENs had the most connectors in Week 4, and the fewest connectors and module hubs in Week 9 (Figure 4; Supplementary Tables S4, S5). That is, aeration led to simplified pMENs over time, possibly because aeration greatly accelerated nutrient consumption or oxidation. Two network hubs were identified: ASV_144 (family *Sphingomonadaceae*) in control Week 9 and ASV_572 (family *Pedosphaeraceae*) in aerated Week 2. No significant correlation was observed for node connectivity among different incubation periods. Furthermore, all the 35 connectors consisted of 35 different nodes, indicating varying among-module node connectivity over time. By contrast, several nodes were module hubs in more than one pMEN, indicating time-steady within-module node connectivity.

### Connectivity of *Desulfobulbaceae*

**Figure 5:**
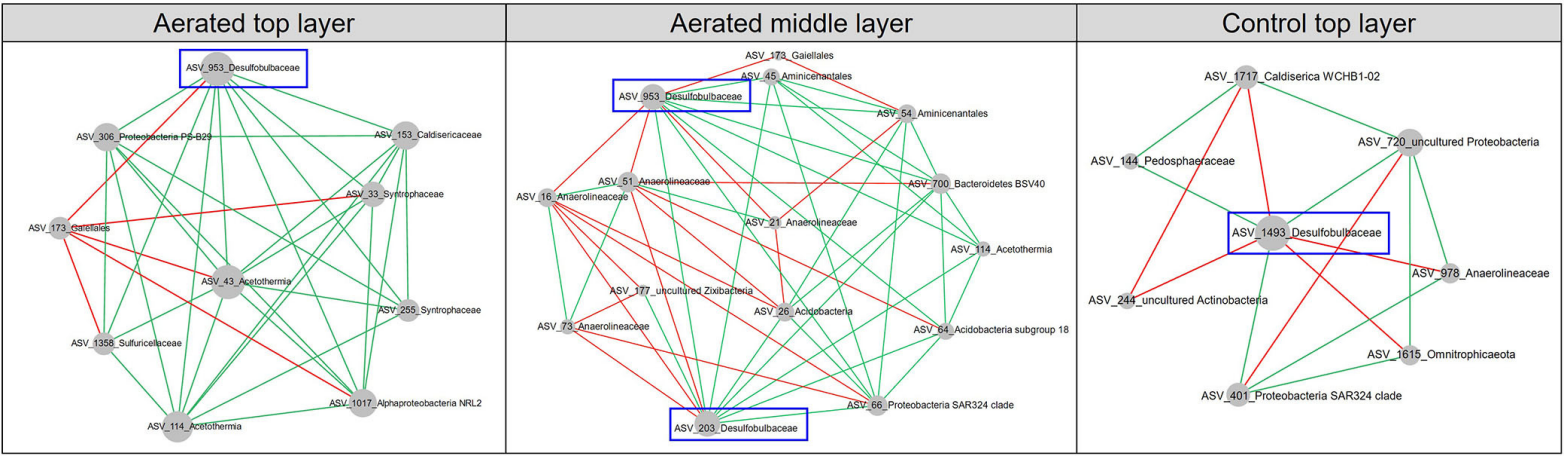
Subnetworks revealing interactions among nodes of *Desulfobulbaceae* and direct-linking nodes of other families in the aerated top, control top, and aerated middle layers. Node size was proportional to node degree in subnetworks. A link represented a significant Spearman's Rho rank correlation between two nodes, green for positive and red for negative correlations.

Aeration had varying impacts on the pMENs. We typically focused on the family *Desulfobulbaceae* which is known for long-distance (~cm) electron transfer between anoxic (i.e., deeper) and oxic (i.e., upper) sediment layers. *Desulfobulbaceae* was detected in the top layers of both control and aerated pMENs but also the middle layer of aerated pMEN (Figure 5). Interestingly, in contrast to the overall simplifying pattern of aerated depth-wise pMENs, nodes of *Desulfobulbaceae* gained increasing connectivity with other groups, particularly at the middle layer. For example, three nodes (ASV_953, ASV_1493, and ASV_203) were members of *Desulfobulbaceae* and established 37 links to other nodes. No discernible patterns were found among these nodes with respect to ASV abundance, taxonomy, or modularity, though all of them were peripherals, and 70% of these links were positive. ASV_953 was present in both aerated top (node degree = 9) and aerated middle (node degree =11) layers, ASV_203 was present in the aerated middle layer (node degree = 11), and ASV_1493 was present in the control top layer with fewer connections (node degree = 7).

**Figure 6:**
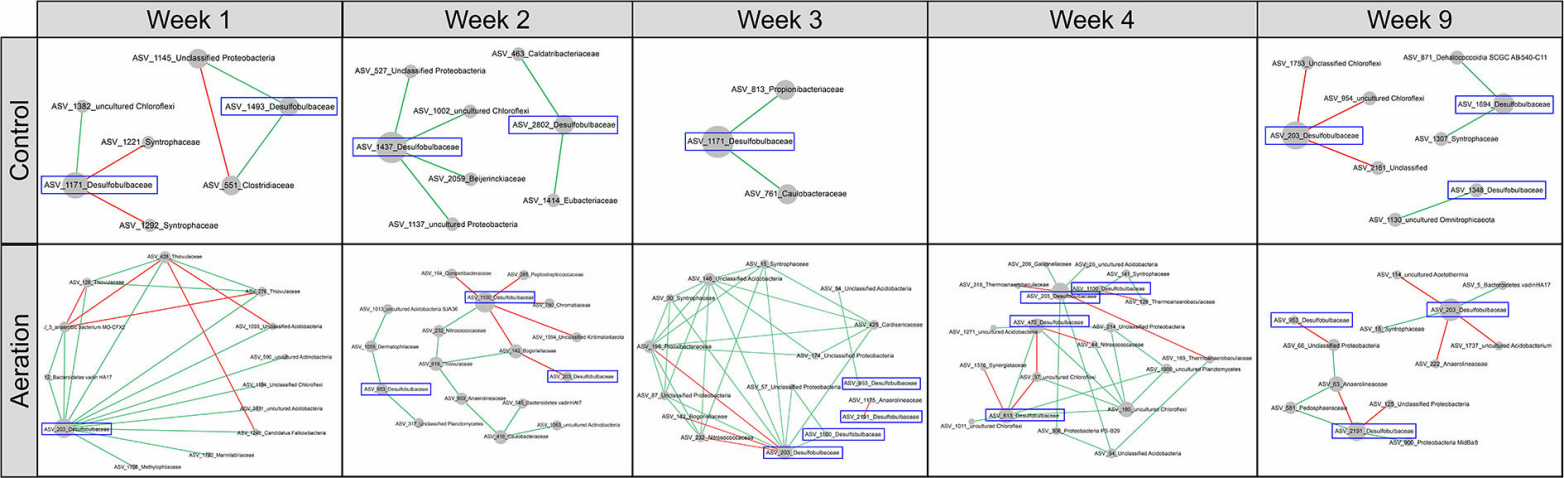
Subnetworks revealing temporal changes in the interactions among nodes of *Desulfobulbaceae* and direct-linking nodes of other families from Week 1 to 9 under aeration or not. *Desulfobulbaceae* was not present in the control Week 4 pMEN. Node size was proportional to node degree in subnetworks. A link represented a significant Spearman's Rho rank correlation between two nodes, green for positive and red for negative correlations.

Meanwhile, 12 nodes in the temporal pMENs were members of *Desulfobulbaceae* and established 80 links to other nodes (Figure 6). Similarly, 70% of these links were positive. Among these 12 nodes, ASV_203 was present in all five temporal-aerated pMENs and functioned as a module hub in Week 1 and Week 4 (Supplementary Table S5), implying its key within-module connectivity under aeration. In contrast, it was absent in control temporal pMENs except at Week 9, with only three negative connections and no significant topological roles. One possible reason might be that aeration led to a niche differentiation within the *Desulfobulbaceae* family.

## Discussion

Though aeration significantly increased DO content at the sediment-water interface, porewater DO became undetectable below ~5 mm in sediment depth. That is, only the partial top layer was exposed to oxygen. However, changes in physiochemical parameters across all layers (0–6 cm) were observed due to aeration, such as the drastic sulfate accumulation and the significant consumption of total carbon and total nitrogen. These changes suggested the existence of biological mediators that extended the surface oxygen impacts down to the middle and deep layers (Nielsen et al., 2010). Noticeably, the subject sediments were characterized as rich in sulfide, especially in the bottom layer (Hu et al., 2021; Wang et al., 2021) but the most intensive sulfide oxidation (i.e., sulfate accumulation) occurred in the middle layer, not the top layer where oxygen was the most abundant, or the bottom layer where sulfide was the most abundant. Both previous studies and our present study found that cable bacteria played a role in bridging the spatially isolated surface oxygen and deep sulfide (Nielsen et al., 2010; Seitaj et al., 2015; Liu et al., 2021).

To visualize changes in microbial interactions due to aeration, we first constructed the depth-wise phylogenetic molecular ecological networks (pMENs) for the aerated and control sediments. All sampling time points were merged so that each group had three pMENs corresponding to the top, middle, and bottom layers. Compared to control pMENs, we observed consistently smaller network sizes and lower average connectivity across all layers of aerated pMENs, suggesting simplified networks (Zhou et al., 2010). When focused on the aerated middle layer where cable bacteria might exist, a higher proportion of positive links, a lower average path length, and a higher average clustering coefficient were observed, suggesting a more cooperative and compact network (Zhou et al., 2010). Aeration also reshaped the pMEN compositions because aerated and control pMENs consisted mostly of different nodes, with only 24.1–31.7% of total nodes shared by two groups. Even among the shared nodes, their degrees and connectivity varied from layer to layer. In addition, aerated middle layer pMEN exhibited unique characteristics among all depth-wise pMENs, seen from (i) the most module hubs and the most keystone nodes, (ii) closer node connections, and (iii) a network hub (ASV_61, *Anaerolineaceae* family, *Chloroflexi* phylum).

We then constructed six temporal pMENs to visualize the changes in microbial covariations over time, in which all three layers at each sampling time point were merged to form one network to represent microbial covariations at the end of Weeks 1, 2, 3, 4, and 9. Aerated and control pMENs were initiated at similar sizes (499 and 517 nodes), but the former kept shrinking over time whereas sizes of the latter remained time-steady, further supporting simplified networks due to aeration. Aerated temporal pMENs also had consistently higher average clustering coefficients and higher average connectivity throughout the incubation. Together, these results indicated simplified but more compact microbial interactions due to aeration, emphasizing greater microbial cooperative behaviors (Zhou et al., 2010).

Environmental perturbations affect microbial interactions by changing the number and connectivity of keystone nodes (i.e., sum of the connector, module hub, and network hub). Without aeration, pMENs had similar numbers of keystone nodes across all layers or across all time points. For depth-wise pMENs, the aerated middle layer was the most complex for having the most connectors and module hubs. For temporal pMENs, they were most complex in Week 4 and Week 9 for having either the most module hubs or the most connectors. Aeration led to decreasing network sizes, meaning fewer nodes were maintaining significant Spearman's correlations above the threshold, possibly because aeration reduced metabolic diversity by suppressing anaerobic metabolisms such as sulfate reduction, denitrification, and methanogenesis, where the terminal electron acceptors are sulfate, nitrate, and carbon dioxide, respectively (Li et al., 2021). The keystone taxa (i.e., taxa of keystone nodes) varied from layer to layer, from time point to time point, and from connectors to module hubs, though connectors were predominantly members of Phyla *Chloroflexi* and *Proteobacteria*, whereas module hubs were predominantly members of Phyla *Chloroflexi, Proteobacteria, Actinobacteria*, and *Verrucomicrobia*. Altogether, aeration altered the connectivity of individual nodes and keystone taxa.

Members of the family *Desulfobulbaceae*, the so-called cable bacteria, could be the possible biological mediators that bridged the spatially isolated surface oxygen and deep sulfide and therefore extended the impacts of surface oxygen to the middle or deep sediment layers. Naturally, cable bacteria in freshwater sediments are the most abundant at the surface and become undetectable below ~2 cm, much deeper into the sediment than DO can reach (Risgaard-Petersen et al., 2015). In this study, cable bacteria were present in the top layers of both control and aerated sediments, but were also present in the aerated middle layer, so that aeration or elevated DO prompt their growth downward the sediment, where sulfide was more abundant than the sediment surface. This explains why the aerated middle layer was where the most intensive sulfur oxidation occurred. In addition, several nodes were identified in both depth-wise and temporal pMENs that were members of *Desulfobulbaceae* or directly connected to *Desulfobulbaceae* nodes or connecting *Desulfobulbaceae* nodes to other microbial groups. The majority (~70%) of these links were positive, suggesting cooperative interactions. Though aerated pMENs were simplified over time, *Desulfobulbaceae* tended to form time-steady, complex, and predominantly positive covariations with other microbial groups, particularly in the middle layer.

## Data availability statement

The datasets presented in this study can be found in online repositories. The names of the repository/repositories and accession number(s) can be found below: https://www.biosino.org/node/, OEP001302.

## Author contributions

ZW, FL, MX, and RQ: experiment design. ZW and EL: data curation and original draft preparation. EL, FL, and YYu: graphics. YYa, MX, and RQ: manuscript review and editing. MX: conceptualization. RQ and MX: funding acquisition and supervision. All authors have read and agreed to submit the current manuscript version for publication.

## Funding

This research was partly supported by Key Realm Research and Development Program of Guangdong Province (2020B0202080001), the National Natural Science Foundation of China (91851202), Guangdong Laboratory for Lingnan Modern Agriculture Project (NT2021010), the Science and Technology Planning Project of Guangdong Province, China (2021B1212040008), and GDAS' Special Project of Science and Technology Development (2022GDASZH-2022010203 and 2022GDASZH-2022010105).

## Conflict of interest

The authors declare that the research was conducted in the absence of any commercial or financial relationships that could be construed as a potential conflict of interest.

## Publisher's note

All claims expressed in this article are solely those of the authors and do not necessarily represent those of their affiliated organizations, or those of the publisher, the editors and the reviewers. Any product that may be evaluated in this article, or claim that may be made by its manufacturer, is not guaranteed or endorsed by the publisher.

## Supplementary material

Supplementary Figure S1 Rarefaction curves of richness **(A)** or Shannon index **(B)** against the sequencing depth.

Supplementary Figure S2 Sulfate accumulation of all layers resulting from aeration. The most intensive sulfate accumulation occurred in the middle layer.

Supplementary Figure S3 Categorized compositions of the microbial community at the phylum level **(A)** and genus level **(B)**. Among the 11 predominant phyla (>1.0% relative abundance), *Proteobacteria* in the top layers showed increased relative abundances in response to aeration throughout the incubation, whereas *Proteobacteria* in the middle and bottom layers did not show significant changes. About 2/3 of the total ASVs did not have an identified genus. Among the 17 most abundant genera (>0.5% relative abundance), *Clostridium* sensu stricto 1, *Romboutsia, Caldisericum*, and *Smithella* were predominant.

Supplementary Figure S4 Non-metric multidimensional scaling analysis of the aerated and control networks at different incubation times. The aerated top layers (black triangles) had the most distinct microbial compositions compared to the other layers throughout the 9-week incubation.

Supplementary Table S1 Summary of node classes shared by aeration and control depth-wise pMENs.

Supplementary Table S2 Connectors in depth-wise pMENs.

Supplementary Table S3 Module hubs in depth-wise pMENs.

Supplementary Table S4 Connectors in temporal pMENs.

Supplementary Table S5 Module hub in temporal pMENs.
