# Supplementary figures and images for: Network analysis reveals microbe-mediated impacts of aeration on deep sediment layer microbial communities
Source: Front Microbiol. 2022 Sep 30;13:931585. doi: 10.3389/fmicb.2022.931585 (PMC9561788; doi:10.3389/fmicb.2022.931585)

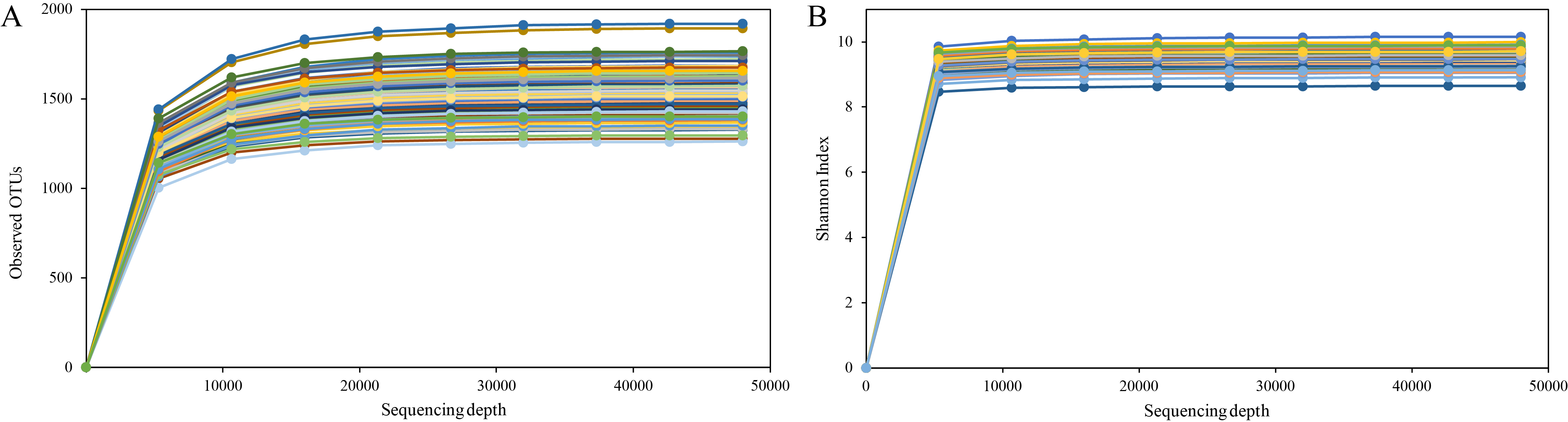

Supplement: Supplementary Figure S1 — Rarefaction curves of richness (A) or Shannon index (B) against the sequencing depth. [file Image_1.JPEG]

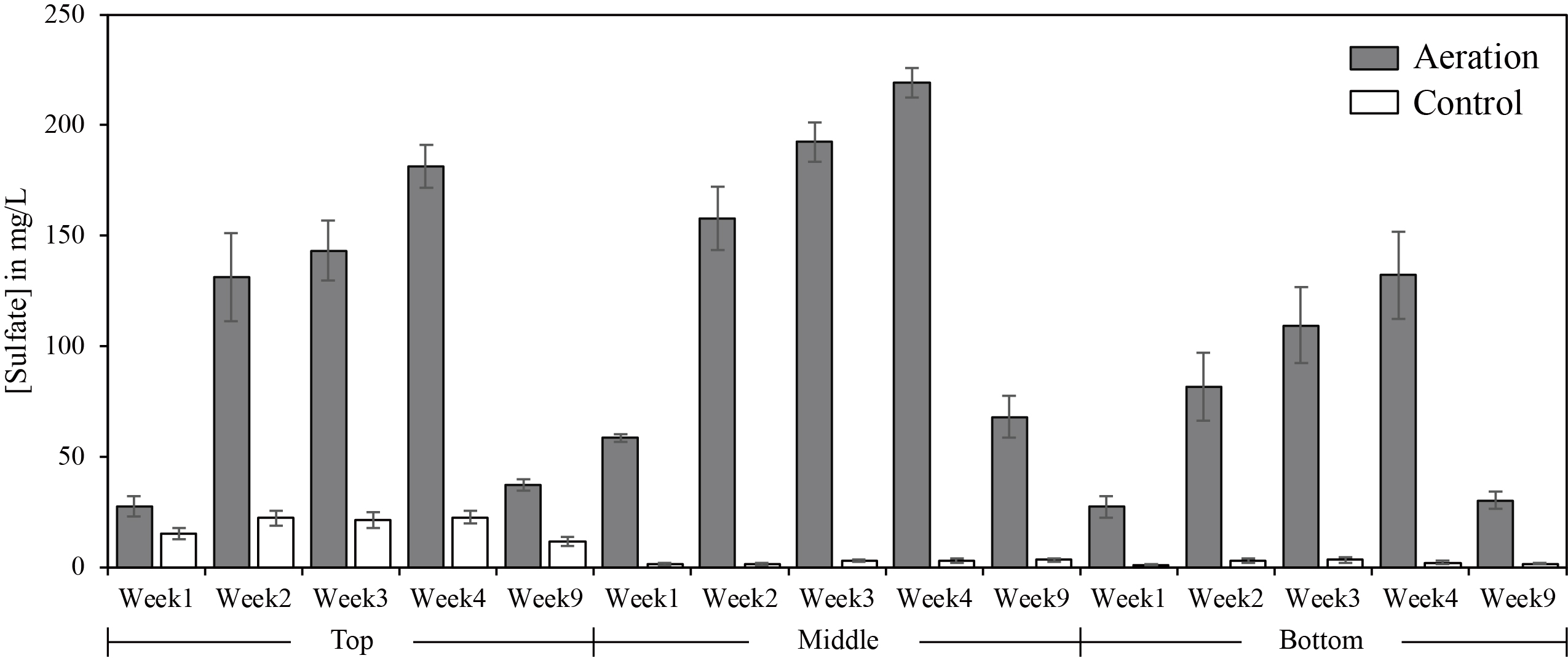

Supplement: Supplementary Figure S2 — Sulfate accumulation of all layers resulting from aeration. The most intensive sulfate accumulation occurred in the middle layer. [file Image_2.JPEG]

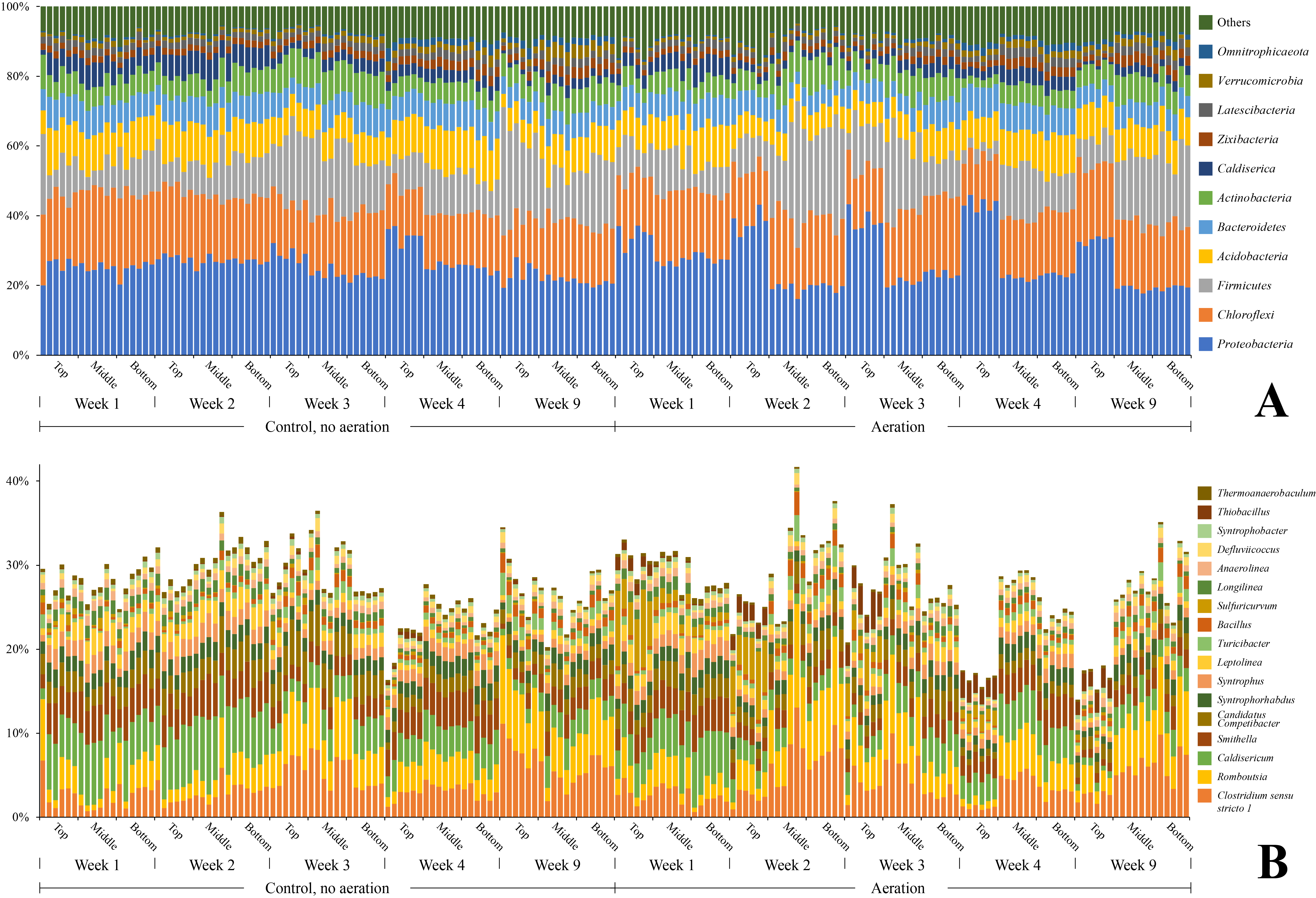

Supplement: Supplementary Figure S3 — Categorized compositions of the microbial community at the phylum level (A) and genus level (B). Among the 11 predominant phyla (>1.0% relative abundance), Proteobacteria in the top layers showed increased relative abundances in response to aeration throughout the incubation, whereas Proteobacteria in the middle and bottom layers did not show significant changes. About 2/3 of the total ASVs did not have an identified genus. Among the 17 most abundant genera (>0.5% relative abundance), Clostridium sensu stricto 1, Romboutsia, Caldisericum, and Smithella were predominant. [file Image_3.JPEG]
