# Supplementary material for: Network analysis reveals microbe-mediated impacts of aeration on deep sediment layer microbial communities
Source: Front Microbiol. 2022 Sep 30;13:931585. doi: 10.3389/fmicb.2022.931585 (PMC9561788; doi:10.3389/fmicb.2022.931585)
Supplement: Supplementary Table S1 — Summary of node classes shared by aeration and control depth-wise pMENs. [file Data_Sheet_1.docx]

**Table S1.** Summary of node classes shared by aeration and control depth-wise pMENs.

| Layer | Class | # nodes | | |
| --- | --- | --- | --- | --- |
|  |  | Aeration | Control | Shared |
| Top | *Chloroflexi* | 68 | 66 | 30 |
|  | *β-Proteobacteria* | 39 | 10 | 6 |
|  | *δ-Proteobacteria* | 33 | 49 | 19 |
|  | *γ-Proteobacteria* | 26 | 20 | 7 |
|  | *Bacteroidetes* | 22 | 25 | 8 |
|  | *Firmicutes* | 20 | 40 | 15 |
|  | *α-Proteobacteria* | 17 | 10 | 4 |
|  | *Acidobacteria* | 15 | 48 | 10 |
|  | *Actinobacteria* | 13 | 22 | 5 |
|  | *Epsilonbacteraeota* | 10 | 1 | 1 |
|  | *Caldiserica* | 9 | 13 | 9 |
|  | *Patescibacteria* | 8 | 4 | 2 |
|  | *Latescibacteria* | 6 | 12 | 4 |
|  | *Zixibacteria* | 3 | 9 | 3 |
|  | *Nitrospirae* | 2 | 1 | 1 |
|  | *Planctomycetes* | 2 | 5 | 1 |
|  | *Spirochaetes* | 2 | 4 | 2 |
|  | Unclassified | 2 | 6 | 2 |
|  | *Verrucomicrobia* | 2 | 6 | 1 |
|  | *Acetohermia* | 2 | 2 | 1 |
|  | *Elusimicrobia* | 1 | 1 | 1 |
| Middle | *Chloroflexi* | 73 | 71 | 35 |
|  | *δ-Proteobacteria* | 50 | 54 | 27 |
|  | *Firmicutes* | 32 | 33 | 16 |
|  | *Acidobacteria* | 27 | 38 | 16 |
|  | *Actinobacteria* | 27 | 27 | 12 |
|  | *Bacteroidetes* | 22 | 22 | 8 |
|  | *Zixibacteria* | 14 | 14 | 8 |
|  | *Caldiserica* | 11 | 9 | 6 |
|  | *Latescibacteria* | 10 | 7 | 4 |
|  | *γ-Proteobacteria* | 8 | 15 | 3 |
|  | *Spirochaetes* | 8 | 7 | 6 |
|  | *α-Proteobacteria* | 7 | 14 | 4 |
|  | *Verrucomicrobia* | 7 | 11 | 4 |
|  | *Patescibacteria* | 5 | 2 | 2 |
|  | *Acetothermia* | 3 | 3 | 3 |
|  | *Planctomycetes* | 3 | 3 | 1 |
|  | *β-Proteobacteria* | 3 | 4 | 1 |
|  | Unclassified | 3 | 6 | 2 |
|  | *Synergistetes* | 2 | 2 | 2 |
|  | *Epsilonbacteraeota* | 1 | 1 | 1 |
|  | *Modulibacteria* | 1 | 1 | 1 |
|  | *Rokubacteria* | 1 | 2 | 1 |

(to be continued)

(Continued)

| Layer | Class | # nodes | | |
| --- | --- | --- | --- | --- |
|  |  | Aeration | Control | Shared |
| Bottom | *Chloroflexi* | 68 | 89 | 47 |
|  | *δ-Proteobacteria* | 39 | 46 | 18 |
|  | *Acidobacteria* | 31 | 45 | 21 |
|  | *Firmicutes* | 29 | 33 | 19 |
|  | *Bacteroidetes* | 17 | 20 | 6 |
|  | *γ-Proteobacteria* | 13 | 21 | 6 |
|  | *Caldiserica* | 12 | 9 | 9 |
|  | *Actinobacteria* | 10 | 22 | 5 |
|  | *α-Proteobacteria* | 6 | 8 | 2 |
|  | *Spirochaetes* | 6 | 5 | 4 |
|  | *Latescibacteria* | 5 | 4 | 1 |
|  | *Omnitrophicaeota* | 5 | 4 | 1 |
|  | *Zixibacteria* | 5 | 15 | 4 |
|  | *Planctomycetes* | 4 | 3 | 2 |
|  | Unclassified | 4 | 4 | 1 |
|  | *Acetothermia* | 3 | 3 | 3 |
|  | *β-Proteobacteria* | 2 | 3 | 1 |
|  | *Verrucomicrobia* | 1 | 8 | 1 |

**Table S2.** Connectors in depth-wise pMENs.

| Group | Layer | ID | *Z_i_* | *P_i_* | Kingdom | Phylum | Class | Order | Family | Genus |
| --- | --- | --- | --- | --- | --- | --- | --- | --- | --- | --- |
| Aeration | Top | ASV_198 | -0.508 | 0.625 | *Bacteria* | *Chloroflexi* | *Dehalococcoidia* | *MSBL5* | Unclassified | Unclassified |
|  |  | ASV_2204 | -0.158 | 0.625 | *Bacteria* | *Proteobacteria* | *Alphaproteobacteria* | *Acetobacterales* | *Acetobacteraceae* | Unclassified |
|  |  | ASV_1411 | -0.773 | 0.666 | *Bacteria* | *Proteobacteria* | *Gammaproteobacteria* | *Betaproteobacteriales* | *Burkholderiaceae* | Unclassified |
|  |  | ASV_266 | 0.465 | 0.666 | *Bacteria* | *Proteobacteria* | *Gammaproteobacteria* | *Oceanospirillales* | *Halomonadaceae* | *Halomonas* |
|  |  | ASV_2182 | -0.021 | 0.653 | *Bacteria* | *Proteobacteria* | *Gammaproteobacteria* | *Xanthomonadales* | *Rhodanobacteraceae* | Unclassified |
|  | Middle | ASV_696 | -0.375 | 0.625 | *Bacteria* | *Acetothermia* | *Acetothermiia* | Unclassified | Unclassified | Unclassified |
|  |  | ASV_516 | -0.104 | 0.625 | *Bacteria* | *Actinobacteria* | *Actinobacteria* | *Propionibacteriales* | *Propionibacteriaceae* | Unclassified |
|  |  | ASV_61 | 2.609 | 0.644 | *Bacteria* | *Chloroflexi* | *Anaerolineae* | *Anaerolineales* | *Anaerolineaceae* | Unclassified |
|  |  | ASV_240 | -0.501 | 0.7 | *Bacteria* | *Chloroflexi* | *Anaerolineae* | *Anaerolineales* | *Anaerolineaceae* | Unclassified |
|  |  | ASV_222 | -0.375 | 0.64 | *Bacteria* | *Chloroflexi* | *Anaerolineae* | *Anaerolineales* | *Anaerolineaceae* | Unclassified |
|  |  | ASV_914 | -0.104 | 0.625 | *Bacteria* | *Firmicutes* | *Clostridia* | *Clostridiales* | *TSAC18* | Unclassified |
|  |  | ASV_808 | -0.471 | 0.666 | *Bacteria* | *Proteobacteria* | *Deltaproteobacteria* | *NKB15* | Unclassified | Unclassified |
|  |  | ASV_492 | -0.104 | 0.722 | *Bacteria* | *Spirochaetes* | *Spirochaetia* | *Spirochaetales* | *Spirochaetaceae* | Unclassified |
|  |  | ASV_191 | -0.195 | 0.68 | *Bacteria* | *Spirochaetes* | *Spirochaetia* | *Spirochaetales* | *Spirochaetaceae* | Unclassified |
|  |  | ASV_95 | -0.48 | 0.625 | *Bacteria* | *Zixibacteria* | Unclassified | Unclassified | Unclassified | Unclassified |
|  | Bottom | ASV_712 | -0.987 | 0.625 | *Bacteria* | *Chloroflexi* | *Anaerolineae* | *Anaerolineales* | *Anaerolineaceae* | Unclassified |
|  |  | ASV_1635 | -0.964 | 0.625 | *Bacteria* | *Chloroflexi* | *Anaerolineae* | *SBR1031* | Unclassified | Unclassified |
|  |  | ASV_341 | -0.577 | 0.625 | *Bacteria* | *Omnitrophicaeota* | Unclassified | Unclassified | Unclassified | Unclassified |
| Control | Top | ASV_920 | -0.449 | 0.625 | *Bacteria* | *Bacteroidetes* | *Bacteroidia* | *Bacteroidales* | Unclassified | Unclassified |
|  |  | ASV_103 | -0.172 | 0.64 | *Bacteria* | *Bacteroidetes* | *Bacteroidia* | *Sphingobacteriales* | *Lentimicrobiaceae* | Unclassified |
|  |  | ASV_1463 | -0.811 | 0.666 | *Bacteria* | *Proteobacteria* | *Deltaproteobacteria* | *Deltaproteobacteria Incertae Sedis* | *Syntrophorhabdaceae* | *Syntrophorhabdus* |
|  | Middle | ASV_2433 | -0.356 | 0.625 | *Bacteria* | *Caldiserica* | *Caldisericia* | *Caldisericales* | *WCHB1-02* | Unclassified |
|  |  | ASV_596 | -0.591 | 0.666 | *Bacteria* | *Chloroflexi* | *Anaerolineae* | *Anaerolineales* | *Anaerolineaceae* | Unclassified |
|  |  | ASV_830 | 1.033 | 0.656 | *Bacteria* | *Chloroflexi* | *Anaerolineae* | *RBG-13-54-9* | Unclassified | Unclassified |
|  |  | ASV_1130 | -0.105 | 0.64 | *Bacteria* | *Omnitrophicaeota* | Unclassified | Unclassified | Unclassified | Unclassified |
|  |  | ASV_1546 | -0.675 | 0.625 | *Bacteria* | *Proteobacteria* | *Gammaproteobacteria* | *Methylococcales* | *Methylococcaceae* | *Candidatus Methylospira* |
|  |  | ASV_894 | -0.105 | 0.625 | *Bacteria* | Unclassified | Unclassified | Unclassified | Unclassified | Unclassified |
|  | Bottom | ASV_712 | -1.127 | 0.75 | *Bacteria* | *Chloroflexi* | *Anaerolineae* | *Anaerolineales* | *Anaerolineaceae* | Unclassified |
|  |  | ASV_1298 | -0.894 | 0.666 | *Bacteria* | Unclassified | Unclassified | Unclassified | Unclassified | Unclassified |

**Table** **S3.** Module hubs in depth-wise pMENs.

| Group | Layer | ID | Z_i_ | P_i_ | Kingdom | Phylum | Class | Order | Family | Genus |
| --- | --- | --- | --- | --- | --- | --- | --- | --- | --- | --- |
| Aeration | Top | ASV_185 | 3.39 | 0.438 | *Bacteria* | *Acinobacteria* | *Acidimicrobiia* | *Actinomarinales* | Unclassified | Unclassified |
|  |  | ASV_414 | 2.777 | 0 | *Bacteria* | *Actnobacteria* | *Actinobacteria* | *Frankiales* | *Cryptosporangiaceae* | *Fodinicola* |
|  |  | ASV_6 | 2.94 | 0 | *Bacteria* | *Caldiserica* | *Caldisericia* | *Caldisericaceae* | *Caldisericales* | *Caldisericum* |
|  |  | ASV_594 | 2.673 | 0 | *Bacteria* | *Chloroflexi* | *Anaerolneae* | *Anaerolineales* | *Anaerolineaceae* | *Leptolinea* |
|  |  | ASV_476 | 2.86 | 0.117 | *Baceria* | *Proteobacteria* | *Alphaprateobacteria* | *Caulobacterales* | *Caulobacteraceae* | *Phenylobacterium* |
|  |  | ASV_416 | 2.99 | 900 | *Bacteria* | *Proteobacteria* | *Alphaprateobacteria* | *Caulobacterales* | *Caulobacteraceae* | *Phenylobacterium* |
|  |  | ASV_220 | 2.729 | 0.064 | *Bacsria* | *Proteobacteria* | *Ganmaproteobacteria* | *Betaproteobacteriales* | *Burkholderiaceae* | *Hydrogenophaga* |
|  |  | ASV_60 | 2.92 | 0 | *Bacteria* | *Proteobacteria* | *Ganmaproteobacteria* | *Betaproteobacteriales* | *Rhodocyclaceae* | *Sulfuorisoma* |
|  |  | ASV_331 | 2.99 | 0.396 | *Baceria* | *Zixibacteria* | Unclassified | Unclassified | Unclassified | Unclassified |
|  | Middle | ASV_20 | 3.023 | 0.386 | *Bacteria* | *Acidobacteria* | *c5LKS83* | *metagenome* | *metagenome* | *metagenome* |
|  |  | ASV_25 | 4.015 | 0.56 | *Baceria* | *Acinobacteria* | *Themoleophilia* | *Gaiellales* | Unclassified | Unclassified |
|  |  | ASV_6 | 2.94 | 0 | *Baceria* | *Caldiserica* | *Caldisericia* | *Caldisericales* | *Caldisericaceae* | *Caldisericum* |
|  |  | ASV_61 | 2.609 | 0.644 | *Baceria* | *Chloroflexi* | *Anasrolineae* | *Anaerolineacene* | *Anaerolineales* | Unclassified |
|  |  | ASV_343 | 5.76 | 0.422 | *Bacteria* | *Chloroflexi* | *Anaerolineae* | *SJA-15* | Unclassified | Unclassified |
|  |  | ASV_663 | 2.717 | 0.277 | *Baceria* | *Verrucomicrobia* | *Verrucomicrobia* | *Pedosphaerales* | *Pedosphaeraceae* | Unclassified |
|  |  | ASV_82 | 2.664 | 0.46 | *Bacteria* | *Zixibacteria* | Unclassified | Unclassified | Unclassified | Unclassified |
|  | Bottom | ASV_137 | 2.748 | 0.461 | *Baceria* | *Acinobacteria* | *Actinobacteria* | *Micrococcales* | *Microbacteriaceae* | *Microbacterium* |
|  |  | ASV_6 | 2.683 | 0.277 | *Bacteria* | *Caldiserica* | *Caldisericia* | *Caldisericales* | *Caldisericaceae* | *Caldisericum* |
|  |  | ASV_63 | 3.24 | 0.095 | *Bac teria* | *Chloroflexi* | *Anaerolineae* | *Anaerolineales* | *Anaerolineaceae* | *RBG-16-58-14* |
|  |  | ASV_79 | 3.185 | 0.539 | *Bacteria* | *Firmicutes* | *Bacilli* | *Bacillales* | *Bacillaceae* | *Bacillus* |
|  |  | ASV_15 | 2.51 | 0 | *Baceria* | *Proteobacteria* | *Deltaproteobacteria* | *Syntrophobacterales* | *Syntrophaceae* | *Smithella* |
|  |  | ASV_44 | 3.471 | 0.234 | *Bacteria* | *Proteobacteria* | *Gammaproteobacteria* | *Nitrosococcales* | *Nitrosococcaceae* | *C175cm.2.12* |

(to be continued)

(continued)

| Group | Layer | ID | Z_i_ | P_i_ | Kingdom | Phylum | Class | Order | Family | Genus |
| --- | --- | --- | --- | --- | --- | --- | --- | --- | --- | --- |
| Control | Top | ASV_278 | 3.575 | 0 | *Bacteria* | *Acidobacteria* | *Thermoanaerobaculia* | *Thermoanaerobaculales* | *Thermoanaerobaculaceae* | *Thermoanaerobaculum* |
|  |  | ASV_28 | 3.102 | 0 | *Bacteria* | *Bacter cidetes* | *Bacteroidia* | *Sphingobacteriales* | *Lentimicrobiaceae* | Unclassified |
|  |  | ASV_995 | 2.828 | 0.444 | *Bacteria* | *Chloroflexi* | *Anaerolineae* | *SBR1031* | Unclassified | Unclassified |
|  |  | ASV_333 | 4.552 | 0 | *Baceria* | *Fimicutes* | *Clostridia* | *Clostridiales* | *Clostridiaceae1* | *Clostridiumsensu stricto 11* |
|  |  | ASV_29 | 2.697 | 0.444 | *Bacteria* | *Latescibacteria* | Unclassified | Unclassified | Unclassified | Unclassified |
|  |  | ASV_913 | 2.829 | 0.37 | *Baceria* | *Planctomicess* | *OM190* | Unclassified | Unclassified | Unclassified |
|  |  | ASV_36 | 2.59 | 0 | *Baceria* | *Proteobacteria* | *Alphaproteobacteria* | *Rhizobiales* | *Xanthobacteraceae* | Unclassified |
|  |  | ASV_127 | 3.102 | 0.214 | *Baceria* | *Proteobacteria* | *Gammaproteobacteria* | *Competibacterales* | *Competibacteraceae* | *Candidatus Competibacter* |
|  |  | ASV_44 | 5.561 | 0 | *Baceria* | *Proteobacteria* | *Gammaproteobacteria* | *Nitrosococcales* | *Nitrosococcaceae* | *C175cm.2.12* |
|  | Middle | ASV_54 | 2.731 | 0 | *Bacteria* | *Acidobacteria* | *Aminicenantia* | *Aminicenantales* | Unclassified | Unclassified |
|  |  | ASV_20 | 4.434 | 0 | *Bacteria* | *Acidobacteria* | *c5LKS83* | *metagenome* | *metagenome* | *metagenome* |
|  |  | ASV_811 | 2.742 | 0.345 | *Baceria* | *Chloroflexi* | *Anaerolineae* | *SBR1031* | Unclassified | Unclassified |
|  |  | ASV_396 | 4.712 | 0.398 | *Baceria* | *Chloroflexi* | *Anasrolineae* | Unclassified | Unclassified | Unclassified |
|  |  | ASV_37 | 2.52 | 0 | *Baceria* | *Chloroflexi* | *Dehalococcoidia* | *MSBL5* | Unclassified | Unclassified |
|  |  | ASV_36 | 2.671 | 0 | *Bacteria* | *Proteobacteria* | *Alphaproteobacteria* | *Rhizobiales* | *Xanthobacteraceae* | Unclassified |
|  |  | ASV_144 | 5.648 | 0.271 | *Baceria* | *Verruconicrobia* | *Verrucomicrobiae* | *Pedosphaerales* | *Pedosphaeraceae* | *ADwrb Bin063-1* |
|  |  | ASV_663 | 4.451 | 0.165 | *Bacteria* | *Verrucomicrobia* | *Verrucomicrobiae* | *Pedosphasrales* | *Pedosphaeraceae* | Unclassified |
|  |  | ASV_395 | 2.723 | 0 | *Bacteria* | *Verruconicrobia* | *Verrucomicrobiae* | *Pedosphasrales* | *Pedosphaeraceae* | Unclassified |
|  |  | ASV_52 | 4.159 | 0.315 | *Bacteria* | *Zixibacteria* | Unclassified | Unclassified | Unclassified | Unclassified |
|  | Bottom | ASV_28 | 4.95 | 0.42 | *Bacteria* | *Bactercidetes* | *Bacteroidia* | *Sphingobacteriales* | *Lentimicrobiaceae* | Unclassified |
|  |  | ASV_14 | 2.786 | 0.18 | *Bacteria* | *Caldiserica* | *Caldisericia* | *Caldisericales* | *Caldisericaceae* | *Caldisericum* |
|  |  | ASV_63 | 3.93 | 0.164 | *Bacteria* | *Chloroflexi* | *Anaerolineae* | *Anaerolineales* | *Anaerolineaceae* | *RBG-16-58-14* |
|  |  | ASV_161 | 2.683 | 0 | *Bacteria* | *Chloroflexi* | *Dehalococcoidia* | *MSBL5* | Unclassified | Unclassified |
|  |  | ASV_33 | 2.91 | 0 | *Bacteria* | *Proteobacteria* | *Deltaproteobacteria* | *Syntrophobacterales* | *Syntrophaceae* | *Smithella* |
|  |  | ASV_144 | 5.197 | 0 | *Bacteria* | *Verrucomicrobia* | *Verrucomicrobiae* | *Pedosphaerales* | *Pedosphaeraceae* | *ADurb.Bin063-1* |

**Table S4.** Connectors in temporal pMENs.

| Group | Time | ID | Z_i_ | P_i_ | Kingdom | Phylum | Class | Order | Family | Genus |
| --- | --- | --- | --- | --- | --- | --- | --- | --- | --- | --- |
| Aeration | Week l | ASV_2511 | 0 | 0.625 | *Bacteria* | *Acidobacteria* | *c5LKS83* | Unclassified | Unclassified | Unclassified |
|  |  | ASV_1411 | 0.283 | 0.641 | *Bacteria* | *Proteobacteria* | *Gammaproteobacteria* | *Betaproteobacteriales* | *Burkholderiacea* | Unclassified |
|  | Week 2 | ASV_701 | 0.143 | 0.625 | *Bacteria* | *Firmicutes* | *Clostridia* | *Clostridiales* | *Clostridiaceae 1* | *Clostridium sensu stricto 9* |
|  |  | ASV_717 | -0.037 | 0.64 | *Bacteria* | *Firmicutes* | *Clostridia* | *Clostridiales* | *Lachnospiraceae* | *Cellulosilyticum* |
|  |  | ASV_834 | 0 | 0.625 | *Bacteria* | *Firmicutes* | *Clostridia* | *Clostridiales* | Unclassified | Unclassified |
|  |  | ASV_572 | 2.728 | 0.642 | *Bacteria* | *Proteobacteria* | *Alphaproteobacteria* | *Sphingomonadales* | *Sphingomonadaceae* | *Novosphingobium* |
|  | Week 3 | ASV_1737 | 0.635 | 0.693 | *Bacteria* | *Acidobacteria* | *Subgroup 18* | Unclassified | Unclassified | Unclassified |
|  |  | ASV_296 | -0.127 | 0.625 | *Bacteria* | *Firmicutes* | *Clostridia* | *Clostridiales* | *Peptostreptococcaceae* | *Terrisporobacter* |
|  |  | ASV_1084 | 0 | 0.625 | *Bacteria* | *Proteobacteria* | *Deltaproteobacteria* | *Deltaproteobacteria Incertae Sedis* | *Syntrophorhabdaceae* | *Syntrophorhabdus* |
|  | Week 4 | ASV_42 | -0.956 | 0.666 | *Bacteria* | *Bacteroidetes* | *Bacteroidia* | *Bacteroidales* | *Bacteroidetes vadinHA17* | Unclassified |
|  |  | ASV_56 | 1.942 | 0.634 | *Bacteria* | *Caldiserica* | *Caldisericia* | *Caidsericales* | *Caldisericaceae* | *Caldisericum* |
|  |  | ASV_53 | -0.349 | 0.72 | *Bacteria* | *Chloroflexi* | *Anaerolineae* | *Anaerolineales* | *Anaerolineaceae* | Unclassified |
|  |  | ASV_3 | 0.094 | 0.641 | *Bacteria* | *Chloroflexi* | *Anaerolineae* | *SBR1031* | *MO-CFX2* | *MO-CFX2* |
|  |  | ASV_85 | -0.848 | 0.625 | *Bacteria* | *Chloroflexi* | *Anaerolineae* | *SBR1031* | Unclassified | Unclassified |
|  |  | ASV_297 | 1.025 | 0.627 | *Bacteria* | *Chloroflexi* | *Anaerolineae* | *SBR1031* | Unclassified | Unclassified |
|  |  | ASV_359 | -0.287 | 0.625 | *Bacteria* | *Firmicutes* | *Bacilli* | *Bacillales* | *Bacillaceae* | *Bacillus* |
|  |  | ASV_525 | 2.45 | 0.622 | *Bacteria* | *Nitrospirae* | *Nitrospira* | *Nitrospirales* | *Nitrospiraceae* | *Nitrospira* |
|  |  | ASV_552 | -0.643 | 0.666 | *Bacteria* | *Proteobacteria* | *Deltaproteobacteria* | *Deltaproteobacteria Incertae Sedis* | *Syntrophorhabdaceae* | *Syntrophorhabdus* |
|  |  | ASV_1638 | -0.225 | 0.64 | *Bacteria* | *Proteobacteria* | *Deltaproteobacteria* | *Sva0485* | Unclassified | Unclassified |
|  | Week 9 | ASV_5 | -0.17 | 0.625 | *Bacteria* | *Bacteroidetes* | *Bacteroidia* | *Bacteroidales* | *Bacteroidetes vadinHA17* | Unclassified |

(to be continued)

(continued)

| Group | Time | ID | Z_i_ | P_i_ | Kingdom | Phylum | Class | Order | Family | Genus |
| --- | --- | --- | --- | --- | --- | --- | --- | --- | --- | --- |
| Control | Week l | ASV_43 | -0.218 | 0.625 | *Bacteria* | *Acetothermia* | *Acetothermiia* | Unclassified | Unclassified | Unclassified bactenium |
|  |  | ASV_332 | 0.709 | 0.66 | *Bacteria* | *Chloroflexi* | *KD4-96* | Unclassified | Unclassified | Unclassified bacte nium |
|  |  | ASV_192 | -0.085 | 0.64 | *Bacteria* | *Proteobacteria* | *Gammaproteobacteria* | *Betaproteobacteriales* | *Nitrosomonadaceae* | *966-1* |
|  | Week 2 | ASV_2511 | 1.105 | 0.666 | *Bacteria* | *Acidobacteria* | *c5LKS83* | Unclassified | Unclassified | Unclassified bacte nium |
|  |  | ASV_68 | 0.345 | 0.625 | *Bacteria* | *Firmicutes* | *Clostridia* | *Clostridiales* | *Clostridiaceae1* | *Clostridium sensu stricto 3* |
|  |  | ASV_2478 | 0.074 | 0.625 | *Bacteria* | *Proteobacteria* | *Alphaproteobacteria* | *Rhizobiales* | *Xanthobacteraceae* | *Bradyrhizobium* |
|  |  | ASV_1042 | -0.518 | 0.64 | *Bacteria* | *Proteobacteria* | *Alphaproteobacteria* | *Rhodospirillales* | *Rhodopirillaceae* | *Defluviicoccus* |
|  | Week 4 | ASV_221 | 0 | 0.625 | *Bacteria* | *Firmicutes* | *Clostridia* | *Clostridiales* | *Clostridiaceae 1* | *Clostridium sensu stricto 1* |
|  |  | ASV_1802 | -0.931 | 0.666 | *Bacteria* | *Proteobacteria* | *Deltaproteobacteria* | *Detaproteobacteria Incertae Sedis* | *Syntrophorhabdaceae* | *Syntrophorhabdus* |
|  | Week 9 | ASV_1789 | -0.936 | 0.625 | *Bacteria* | *Omnitrophicaeota* | Unclassified | Unclassified | Unclassified | Unclassified |
|  |  | ASV_662 | -0.936 | 0.666 | *Bacteria* | *Verrucomicrobia* | *Verrucomicrobiae* | *Pedosphaerales* | *Pedosphaeraceae* | *ADurb.Bin063-1* |
|  |  | ASV_144 | 2.588 | 0.639 | *Bacteria* | *Verrucomicrobia* | *Verrucomicrobiae* | *Pedosphaerales* | *Pedosphaeraceae* | *ADurb.Bin063-1* |
|  |  | ASV_2265 | 0.189 | 0.635 | *Bacteria* | *Verrucomicrobia* | *Verrucomicrobiae* | *Pedosphaerales* | *Pedosphaeraceae* | Undlassified |
|  |  | ASV_504 | 0.979 | 0.625 | *Bacteria* | *Zixibacteria* | Unclassified | Unclassified | Unclassified | Unclassified |
|  |  | ASV_388 | -0.182 | 0.625 | *Bacteria* | *Zixibacteria* | Unclassified | Unclassified | Unclassified | Unclassified |

**Table S5.** Module hub in temporal pMENs.

| **Group** | **Time** | **ID** | ***Z_i_*** | ***P_i_*** | **Kingdom** | **Phylum** | **Class** | **Order** | **Family** | **Genus** |
| --- | --- | --- | --- | --- | --- | --- | --- | --- | --- | --- |
| **Aeration** | **Week l** | ASV_148 | 2.700 | 0.076 | *Bacteria* | *Acidobacteria* | *Aminicenantia* | *Aminicenantales* | Unclassified | Unclassified |
|  |  | ASV_26 | 2.700 | 0.197 | *Bacteria* | *Acidobacteria* | *c5LKS83* | Unclassified | Unclassified | Unclassified |
|  |  | ASV_5 | 2.751 | 0 | *Bacteria* | *Bacteroidetes* | *Bacteroidia* | *Bacteroidales* | *Bacteroidetes vadinHA17* | Unclassified |
|  |  | ASV_21 | 2.588 | 0.218 | *Bacteria* | *Chloroflexi* | *Anaerolineae* | *Anaerolineales* | *Anaerolineaceae* | Unclassified |
|  |  | ASV_276 | 2.866 | 0.209 | *Bacteria* | *Epsilonbacteraeota* | *Campylobacteria* | *Campylobacterales* | *Thiovulaceae* | *Sulfuricurvum* |
|  |  | ASV_428 | 2.659 | 0.355 | *Bacteria* | *Epsilonbacteraeota* | *Campylobacteria* | *Campylobacterales* | *Thiovulaceae* | *Suifuricurvum* |
|  |  | ASV_340 | 4.145 | 0 | *Bacteria* | *Firmicutes* | *Bacillt* | *Bacillales* | *Bacillaceae* | *Bacillus* |
|  |  | ASV_203 | 2.751 | 0.486 | *Bacteria* | *Proteobacteria* | *Deltaproteobacteria* | *Desulfobacterales* | *Desulfobulbaceae* | *Candidatus Electronema* |
|  | **Week 2** | ASV_298 | 2.765 | 0.277 | *Bacteria* | *Chloroflexi* | *Anaerolineae* | *SJA-15* | Unclassified | Unclassified |
|  |  | ASV_113 | 2.560 | 0 | *Bacteria* | *Chloroflexi* | *Dehalococcoidia* | *MSBL5* | Unclassified | Unclassified |
|  |  | ASV_126 | 3.348 | 0.257 | *Bacteria* | *Epsilonbacteraeota* | *Campylobacteria* | *Campylobacterales* | *Thiovulaceae* | *Sulfuricurvum* |
|  |  | ASV_1501 | 2.898 | 0.512 | *Bacteria* | *Firmicutes* | *Clostridia* | *Clostridiales* | *Clostridiaceae 1* | *Hathewaya* |
|  |  | ASV_416 | 3.485 | 0.520 | *Bacteria* | *Proteobacteria* | *Alphaproteobacteria* | *Caulobacterales* | *Caulobacteraceae* | *Phenylobacterium* |
|  |  | ASV_626 | 2.919 | 0.556 | *Bacteria* | *Proteobacteria* | *Alphaproteobacteria* | *Sphingomonadales* | *Sphingomonadaceae* | *Novosphingobium* |
|  |  | ASV_572 | 2.728 | 0.642 | *Bacteria* | *Proteobacteria* | *Alphaproteobacteria* | *Sphingomonadales* | *Sphingomonadaceae* | *Novosphingobium* |
|  |  | ASV_646 | 3.190 | 0.534 | *Bacteria* | *Proteobacteria* | *Gammaproteobacteria* | *Betaproteobacteriales* | *Chromobacteriaceae* | *Vogesella* |
|  |  | ASV_60 | 2.716 | 0.299 | *Bacteria* | *Proteobacteria* | *Gammaproteobacteria* | *Betaproteobacteriales* | *Rhodocyclaceae* | *Sulfurisoma* |
|  |  | ASV_275 | 3.190 | 0.387 | *Bacteria* | *Verrucomicrobia* | *Verrucomicrobiae* | *Chthoniobacterales* | *Xiphinemato bacteraceae* | *Candidatus Xiphinematobacter* |
|  | **Week 3** | ASV_10 | 2.716 | 0 | *Bacteria* | *Caldiserica* | *Caldisericia* | *Caldisericales* | *Caldsericaceae* | *Caldisericum* |
|  |  | ASV_18 | 2.705 | 0.320 | *Bacteria* | *Chioroflexi* | *Anaerolineae* | *Anaerolineales* | *Anaerolneaceae* | *Leptolinea* |
|  |  | ASV_174 | 2.922 | 0 | *Bacteria* | *Proteobacteria* | *Deltaproteobacteria* | *SAR324 clade* | Unclassifed | Unclassified |
|  |  | ASV_209 | 2.510 | 0.253 | *Bacteria* | *Proteobacteria* | *Gammaproteobacteria* | *Betaproteobacteriales* | *Gallionellaceae* | *Gallionella* |
|  |  | ASV_266 | 3.320 | 0 | *Bacteria* | *Proteobacteria* | *Gammaproteobacteria* | *Oceanospirillales* | *Halomonadaceae* | *Halomonas* |
|  | **Week 4** | ASV_180 | 2.788 | 0 | *Bacteria* | *Chioroflexi* | *Dehalococcoidia* | *MSBL5* | Unclassified | Unclassified |
|  |  | ASV_2 | 2.591 | 0.119 | *Bacteria* | *Firmicutes* | *Clostridia* | *Clostridiales* | *Clostridiaceae 1* | *Clostridium sensu stricto 1* |
|  |  | ASV_1 | 2.683 | 0.042 | *Bacteria* | *Firmicutes* | *Clostridia* | *Clostridiales* | *Peptostreptococcaceae* | *Romboutsia* |
|  |  | ASV_744 | 3.444 | 0.180 | *Bacteria* | *Proteobacteria* | *Alphaproteobacteria* | *Dongiales* | *Dongiaceae* | *Dongia* |
|  |  | ASV_1315 | 5.196 | 0.537 | *Bacteria* | *Proteobacteria* | *Alphaproteobacteria* | *Dongiales* | *Dongiaceae* | *Dongia* |
|  |  | ASV_203 | 3.013 | 0 | *Bacteria* | *Proteobacteria* | *Deltaproteobacteria* | *Desulfobacterales* | *Desulfobulbaceae* | *Candidatus Electronema* |
|  | **Week 9** | ASV_6 | 2.551 | 0.448 | *Bacteria* | *Caldiserica* | *Caldisericia* | *Caldisericales* | *Caldisericaceae* | *Caldisericum* |
|  |  | ASV_63 | 2.804 | 0 | *Bacteria* | *Chloroflexi* | *Anaerolineae* | *Anaerolineales* | *Anaerolineaceae* | *RBG-16-58-14* |
|  |  | ASV_122 | 2.551 | 0.277 | *Bacteria* | *Latescibacteria* | PRR-10 | PRR-10 | PRR-10 | PRR-10 |
| **Control** | **Week l** | ASV_148 | 2.505 | 0.375 | *Bacteria* | *Acidobacteria* | *Aminicenantia* | *Aminicenantales* | Unclassified | Unclassified |
|  |  | ASV_881 | 3.142 | 0 | *Bacteria* | *Actinobacteria* | *Thermoleophilia* | *Gaiellales* | Unclassified | Unclassified |
|  |  | ASV_463 | 2.985 | 0.406 | *Bacteria* | *Atribacteria* | *Caldatribacteriia* | *Caldatribacteriales* | *Caldatribacteriaceae* | *Candidatus Caldatribacterium* |
|  |  | ASV_18 | 2.656 | 0 | *Bacteria* | *Chloroflexi* | *Anaerolineae* | *Anaerolineales* | *Anaerolneaceae* | *Leptolinea* |
|  |  | ASV_567 | 3.142 | 0 | *Bacteria* | *Proteobacteria* | *Gammaproteobacteria* | *Cellvibrionales* | *Spongiibacteraceae* | *BD1-7 clade* |
|  |  | ASV_831 | 2.501 | 0 | *Bacteria* | *Proteobacteria* | *Deltaproteobacteria* | *Myxococcales* | *PS-B29* | Unclassified |
|  |  | ASV_191 | 3.044 | 0 | *Bacteria* | *Spirochaetes* | *Spirochaetia* | *Spirochaetales* | *Spirochaetaceae* | Unclassified |
|  |  | ASV_1292 | 2.520 | 0 | *Bacteria* | *Proteobacteria* | *Deltaproteobacteria* | *Syntrophobacterales* | *Syntrophaceae* | Unclassified |
|  | **Week 2** | ASV_148 | 2.505 | 0.375 | *Bacteria* | *Acidobacteria* | *Aminicenantia* | *Aminicenantales* | Unclassified | Unclassified |
|  |  | ASV_164 | 2.505 | 0 | *Bacteria* | *Acidobacteria* | *c5LKS83* | Unclassified | Unclassified | Unclassified |
|  |  | ASV_2045 | 3.000 | 0 | *Bacteria* | *Bacteroidetes* | *Bacteroidia* | *Bacteroidales* | *Bacteroidetes vadinHA17* | Unclassified |
|  |  | ASV_1345 | 3.196 | 0 | *Bacteria* | *Chloroflexi* | *Anaerolineae* | *SBR1031* | Unclassified | Unclassified |
|  |  | ASV_457 | 2.990 | 0.244 | *Bacteria* | *Chloroflexi* | *Anaerolineae* | *SJA-15* | Unclassified | Unclassified |
|  |  | ASV_345 | 2.624 | 0 | *Bacteria* | *Firmicutes* | *Clostridia* | *Clostridiales* | *Clostridiaceae 1* | *Clostridium sensu stricto 1* |
|  |  | ASV_1845 | 2.877 | 0.446 | *Bacteria* | *Firmicutes* | *Clostridia* | *Clostridiales* | *Family XIII* | *Anaerovorax* |
|  |  | ASV_2059 | 2.603 | 0 | *Bacteria* | *Proteobacteria* | *Alphaproteobacteria* | *Rhizobiales* | *Beijerinckiaceae* | *alphal cluster* |
|  |  | ASV_831 | 2.501 | 0 | *Bacteria* | *Proteobacteria* | *Deltaproteobacteria* | *Myxococcales* | *PS-B29* | Unclassified |
|  | **Week 3** | ASV_637 | 2.921 | 0.375 | *Bacteria* | *Acidobacteria* | *Acidobacteriia* | *Solibacterales* | *Solibacteraceae* | *Bryobacter* |
|  |  | ASV_14 | 3.713 | 0.410 | *Bacteria* | *Caldiserica* | *Caldisericia* | *Caldisericales* | *Caldisericaceae* | *Caldisericum* |
|  |  | ASV_2 | 2.554 | 0 | *Bacteria* | *Firmicutes* | *Clostridia* | *Clostridiales* | *Clostridiaceae 1* | *Clostridium sensu stricto 1* |
|  |  | ASV_1274 | 2.755 | 0 | *Bacteria* | *Firmicutes* | *Clostridia* | *Clostridiales* | *Clostridiaceae 1* | *Clostridium sensu stricto 2* |
|  |  | ASV_793 | 3.366 | 0 | *Bacteria* | *Firmicutes* | *Clostridia* | *Clostridiales* | *Lachnospiraceae* | *Epulopiscium* |
|  |  | ASV_1 | 2.554 | 0 | *Bacteria* | *Firmicutes* | *Clostridia* | *Clostridiales* | *Peptostreptococcaceae* | *Romboutsia* |
|  |  | ASV_2972 | 3.157 | 0 | *Bacteria* | *Patescibacteria* | *Microgenomatia* | *Candidatus Woesebacteria* | Unclassified | Unclassified |
|  | **Week 4** | ASV_438 | 2.520 | 0.531 | *Bacteria* | *Bacteroidetes* | *Bacteroidia* | *Bacteroidales* | *SB-5* | Unclassified soil bacterium |
|  |  | ASV_398 | 2.888 | 0.421 | *Bacteria* | *Epsilonbacteraeota* | *Campylobacteria* | *Campylobacterales* | *Thiovulaceae* | *Sulfuricurvum* |
|  |  | ASV_1809 | 3.619 | 0.244 | *Bacteria* | *Omnitrophicaeota* | Unclassified | Unclassified | Unclassified | Unclassified |
|  |  | ASV_1881 | 3.181 | 0.277 | *Bacteria* | *Planctomycetes* | *OM190* | Unclassified | Unclassified | Unclassified |
|  |  | ASV_120 | 3.884 | 0.296 | *Bacteria* | *Proteobacteria* | *Magnetococcia* | *Magnetococcales* | *Magnetococcaceae* | *Magnetococcus* |
|  |  | ASV_2066 | 2.520 | 0 | *Bacteria* | *WS2* | Unclassified | Unclassified | Unclassified | Unclassified |
|  | **Week 9** | ASV_1089 | 2.759 | 0 | *Bacteria* | *Bacteroidetes* | *Bacteroidia* | *Bacteroidales* | *Bacteroidetes vadinHA17* | Unclassified Cytophagales |
|  |  | ASV_118 | 2.832 | 0 | *Bacteria* | *Chloroflexi* | *OLB14* | Unclassified | Unclassifed | Unclassified |
|  |  | ASV_110 | 2.588 | 0.244 | *Bacteria* | *Epsilonbacteraeota* | *Campylobacteria* | *Campylobacterales* | *Thiovulaceae* | *Sulfuricurvum* |
|  |  | ASV_71 | 2.795 | 0.497 | *Bacteria* | *Proteobacteria* | *Deltaproteobacteria* | *Syntrophobacterales* | *Syntrophaceae* | Unclassified |
|  |  | ASV_1139 | 5.980 | 0.226 | *Bacteria* | *Proteobacteria* | *Gammaproteobacteria* | *Betaproteobacteriales* | *Burkholderiaceae* | *Limnobacter* |
|  |  | ASV_144 | 2.588 | 0.639 | *Bacteria* | *Verrucomicrobia* | *Verrucomicrobiae* | *Pedosphaerales* | *Pedosphaeraceae* | *ADurb.Bin063-1* |
|  |  | ASV_3941 | 2.583 | 0 | *Bacteria* | *Verrucomicrobia* | *Verrucomicrobiae* | *Pedosphaerales* | *Pedosphaeraceae* | *ADurb.Bin063-1* |
